# An in vivo selection system with tightly regulated gene expression enables directed evolution of highly efficient enzymes

**DOI:** 10.1038/s41598-021-91204-4

**Published:** 2021-06-03

**Authors:** Parinthon Nearmnala, Manutsawee Thanaburakorn, Watanalai Panbangred, Pimchai Chaiyen, Narupat Hongdilokkul

**Affiliations:** 1 Department of Biotechnology, Faculty of Science, Mahidol University, Bangkok, 10400 Thailand; 2 Mahidol University-Osaka University Collaborative Research Center for Bioscience and Biotechnology (MU-OU:CRC), Faculty of Science, Mahidol University, Bangkok, 10400 Thailand; 3 School of Biomolecular Science and Engineering, Vidyasirimedhi Institute of Science and Technology (VISTEC), Rayong, 21210 Thailand

**Keywords:** Molecular engineering, Experimental evolution, Molecular evolution, Enzymes, Molecular biology

## Abstract

In vivo selection systems are powerful tools for directed evolution of enzymes. The selection pressure of the systems can be tuned by regulating the expression levels of the catalysts. In this work, we engineered a selection system for laboratory evolution of highly active enzymes by incorporating a translationally suppressing *cis* repressor as well as an inducible promoter to impart stringent and tunable selection pressure. We demonstrated the utility of our selection system by performing directed evolution experiments using TEM β-lactamase as the model enzyme. Five evolutionary rounds afforded a highly active variant exhibiting 440-fold improvement in catalytic efficiency. We also showed that, without the *cis* repressor, the selection system cannot provide sufficient selection pressure required for evolving highly efficient TEM β-lactamase. The selection system should be applicable for the exploration of catalytic perfection of a wide range of enzymes.

## Introduction

Genetic selection is a powerful tool for directed evolution of macromolecular catalysts^1–4^. By coupling the activity of the enzyme of interest (EOI) with the survival of a host organism, in vivo selection allows simple and robust identification of improved EOI variants. In this approach, mutagenic library members of the gene of interest (GOI) are expressed in host cells that require the function of EOI to survive and replicate under specific experimental conditions. Among these cells, most of which express protein variants containing deleterious or neutral mutations, while beneficial mutations are only found in a small subpopulation. A selection system plays a crucial role in differentiating improved clones from those with impaired or unaltered activity by providing a growth advantage only to the former.

In vivo selection systems are particularly useful for evolving enzymes with higher catalytic activity or altered substrate specificity^5–16^. Such systems can be used when host cell viability depends on the catalyzed reactions of the enzymes to produce essential metabolites or degrade toxic compounds. Since the rate of enzyme-catalyzed reactions in living cells is dependent on the kinetic parameters and the concentration of EOIs, selection pressure for higher catalytic efficiency can be increased by decreasing enzyme expression levels^7,9,13,15^. Traditionally, in *E. coli*, this can be achieved by exchanging a constitutive promoter with a weaker promoter or by subcloning GOI to a plasmid with a lower copy number^13,15^. Nevertheless, the discrete levels of gene expression regulated by these genetic elements render in vivo selection cumbersome because an array of promoters and origins of replication must be tested to evaluate the growth of host cells under different enzyme expression levels.

**Figure 1:**
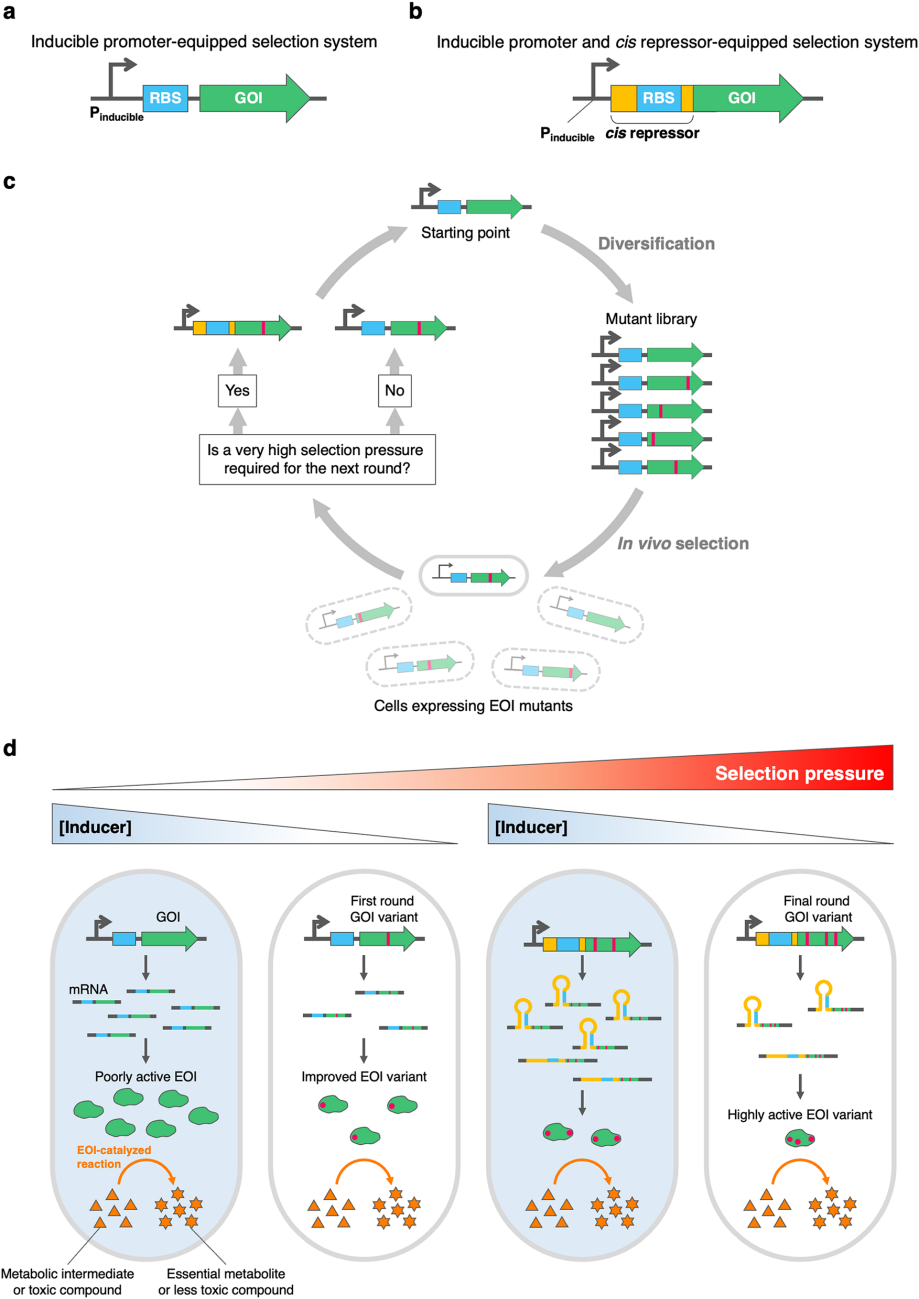
Overview of in vivo selection systems with tunable selection pressure. (**a**) Inducible promoter-equipped selection system. (**b**) Inducible promoter and *cis* repressor-equipped selection system. P_inducible_, inducible promoter; RBS, ribosome binding site; GOI, Gene of interest. In both selection systems, GOI is placed downstream of the inducible promoter. (**c**) General schematic of the selection-based directed evolution experiment conducted in this study. First, GOI is subjected to random mutagenesis to create a mutant library. Bacterial cells expressing EOI mutants are then cultivated under selective conditions, in which only improved mutants can grow. If the (**a**) selection system cannot provide higher selection pressure for the next evolutionary round, the (**b**) selection system is used instead. (**d**) The regulation of selection pressure of the (**a**) and (**b**) selection systems. The survival of cells is coupled to the catalytic activity of EOI. Selection pressure of the selection systems can be modulated by adjusting the concentration of the expression inducer. The EOI starting point requires a high level of expression to catalyze the growth-limiting reaction above a certain rate threshold. Improved EOI variants can be selected by reducing the EOI expression level. The (**b**) selection system can be used when the (**a**) selection system can no longer provide sufficient selection pressure.

To simplify in vivo selection, inducible promoters, such as tetracycline promoter (P_tet_), can be used to regulate the expression of EOIs (Fig. 1)^7,9^. Selection pressure can be tuned by adjusting the amounts of the expression inducers. For sluggish enzymes, large amounts of inducers must be supplemented to allow host organisms to grow. The selection of evolved variants can then be performed by lowering inducer concentration such that their parental clones do not grow.

Although such selection strategies have been used for evolving many enzymes in *E. coli*, the extent to which these catalysts can be evolved is limited by inadequate selection pressure of the selection systems as a result of leaky gene expression. For example, when P_tet_ is used, modestly active enzymes can already confer growth to auxotrophic *E. coli* strains even when no inducer is added to the growth media^9^. Thus, the lack of high selection stringency is a major bottleneck of directed evolution of enzymes towards high catalytic activity. To further increase selection pressure, protein degradation tags (e.g. SsrA and RepA tags), which lead tagged proteins to the proteolytic machinery of *E. coli*^17,18^, can be appended to the termini of enzymes^7,9^. However, the C-terminal SsrA tag is prone to selecting false positives in which nonsense and frameshift mutations in close proximity to the C-terminus delete the tag. The N-terminal RepA tag is not compatible with N-terminal secretion signal sequences, which are important for periplasmic and extracellular protein expression.

To overcome these shortcomings, we aimed to devise a novel tool for in vivo selection by incorporating the transcriptional regulation of P_tet_ with a translationally suppressible element. We rationalized that RNA-based parts might be suitable for this application because, unlike protein degradation tags, genetic elements based on RNA sequence and structure are amenable to response tuning and should be applicable to a broad host range. *Cis*-acting translational repressors, or *cis* repressors, are short 5′ untranslated segments of mRNAs that form ribosome binding site-sequestering hairpin structures^19^. Since these genetic parts have been utilized as a synthetic biology tool for downregulating gene expression in many organisms^20–22^, we expected that a *cis* repressor might be able to extend the dynamic range of P_tet_ and enable directed evolution of highly active enzymes (Fig. 1).

Here, we present the construction and optimization of an in vivo selection system based on the above-mentioned idea. We further demonstrate a proof-of-concept directed evolution experiment in which a crippled TEM β-lactamase variant containing an active site mutation was initially evolved in the P_tet_-equipped selection system and afterwards in the system that combines both P_tet_ and a *cis* repressor for higher selection stringency. Our results showed that, together with P_tet_, the *cis* repressor imparted tightly regulated tunable gene expression, which allowed the evolution of a highly active TEM variant that still retains the active site mutation. We believe that in vivo selection systems exploited in this study will enable selection-based directed evolution of a wide range of highly efficient enzymes.

## Results

### The dynamic range of P_tet_

Previous in vivo selection systems in which randomized libraries of GOI variants were cloned downstream of P_tet_ were successfully employed for directed evolution of enzymes in *E. coli*^7,9^. The selection pressure of a P_tet_-based system can be modulated by adjusting the concentration of tetracycline or anhydrotetracycline (aTc), which is the inducer of P_tet_, to regulate the intracellular concentration of EOI. Since host cells require a biochemical reaction to be catalyzed above a certain rate to survive and grow, such a selection system can facilitate a directed evolution experiment by restricting the intracellular concentration of EOI at a level that permits growth under selective conditions to only improved EOI variants. Through repeated cycles of directed evolution, the selection pressure of the P_tet_-based system can be increased by lowering the concentration of the inducer until one of the following scenarios is reached: (1) evolved EOI variants that achieve the catalytic goal are obtained, (2) further amino acid substitutions can no longer improve the kinetics or stability of evolved EOI variants, or (3) higher selection stringency conditions cannot be applied.

**Figure 2:**
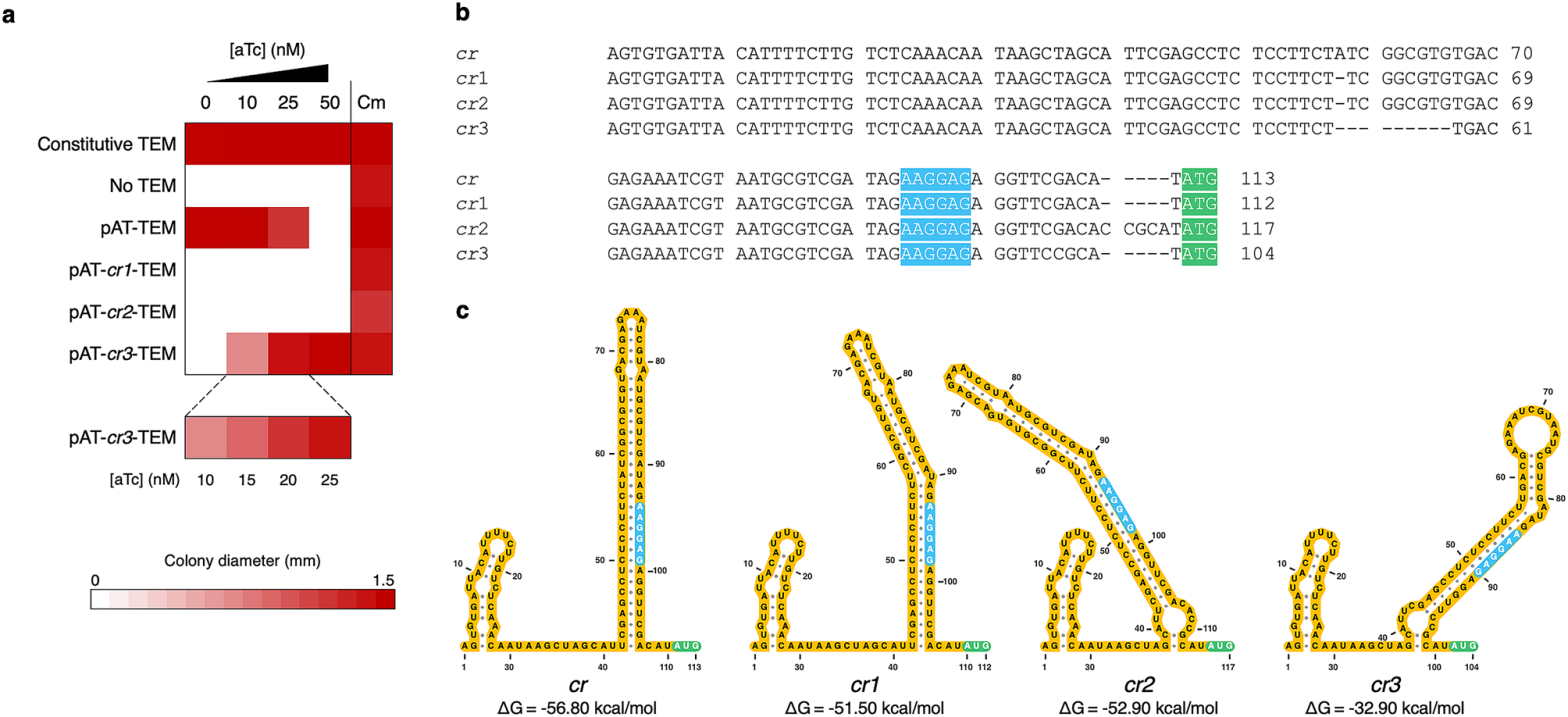
Tuning the dynamic range of the in vivo selection system. (**a**) Heat map representing the average colony diameter of *E. coli* XL1-Blue expressing the wild-type TEM under the control of different expression vectors. Each TEM-expressing *E. coli* strain was grown on LB agar plates containing 50 µg/ml ampicillin (LB Amp50) and varying concentrations of aTc as well as on LB agar plates containing 30 µg/ml chloramphenicol (LB Cm30) at 37 °C for 24 h. *E. coli* XL1-Blue pAT containing no TEM gene and *E. coli* XL1-Blue pAT-TEM_c_, which constitutively expresses TEM, were used as the negative and positive controls, respectively. Colony diameters were determined by ImageJ^25^. The raw data are shown in Table S1. (**b**) The sequence alignment of the original *cis* repressor and its mutants. (**c**) *Cis* repressor secondary structures predicted by Mfold^26^. Ribosome binding sites and start codons are highlighted in blue and green, respectively. The graphical representation of *cis* repressor structures was generated by VARNA^27^.

To test whether P_tet_ alone can provide sufficient selection pressure for selecting highly active catalysts in *E. coli*, we chose TEM β-lactamase (TEM) as the model enzyme because its kinetics is partially diffusion-limited^23^ and its β-lactam antibiotic-degrading activity allows a simple selection regime^24^. We constructed a selection vector for TEM by replacing the TEM gene in the pKTR-mMjCMneg plasmid^7^ with a chloramphenicol resistance gene and subsequently cloning the wild-type TEM gene downstream of P_tet_ between the *Nde*I and *Xho*I sites. The resulting plasmid, designated pAT-TEM, was then used to transform *E. coli* XL1-Blue. The dynamic range of P_tet_ was evaluated by performing a growth assay in which the colony size of XL1-Blue/pAT-TEM growing on LB agar plates containing 50 µg/ml ampicillin (LB Amp50) and varying aTc concentrations was measured. Even without aTc, the basal expression level of P_tet_ allowed XL1-Blue/pAT-TEM to grow at a comparable rate to the *E. coli* strain that constitutively expressed the wild-type TEM. At 50 nM aTc, no growth was observed for XL1-Blue/pAT-TEM, probably because very high expression level of TEM is toxic to *E. coli* (Fig. 2a). As the selection step of directed evolution requires selective conditions in which parental genes cannot confer a growth advantage to the host cells. This high growth rate of XL1-Blue/pAT-TEM under the highest stringency conditions highlighted the limitation of P_tet_ on evolving highly efficient enzymes.

### Equipping the in vivo selection system with a *cis* repressor

In order to overcome the limitation of P_tet_, we next sought to extend the dynamic range of the selection system by incorporating a translationally suppressing *cis* repressor to reduce the basal gene expression level^22^. We hypothesized that reducing both induced and uninduced expression levels can shift the dynamic range of P_tet_ towards a lower expression range. To test this idea, a *cis* repressor sequence, named *cr,* was incorporated into pAT-TEM between P_tet_ and the TEM gene to generate pAT-*cr*-TEM. The *cr* repressor was previously designed to be part of the 5′ untranslated region of mRNA and form a hairpin structure that sequesters the ribosome binding site of the gene downstream, thereby decreasing the translational level^22^. In multiple attempts to construct pAT-*cr*-TEM, we failed to obtain the correct sequence of the *cis* repressor. The DNA sequencing results of eight clones showed different patterns of nucleotide insertions and deletions (Figure S1), which were probably caused by the formation of extensive secondary structure of *cr* during PCR^28^. Nevertheless, two *cis* repressor mutants, designated *cr1* and *cr2*, which showed highest sequence similarity to *cr* (Fig. 2b), were chosen for dynamic range characterization by the growth assay. The result showed that XL1-Blue/pAT-*cr1*-TEM and XL1-Blue/pAT-*cr2*-TEM did not grow on LB Amp50 at any concentrations of aTc (Fig. 2a). The complete suppression of TEM expression by *cr1* and *cr2* was likely to be attributed to the formation of stable mRNA hairpin structures that fully inhibit the translation of the TEM gene (Fig. 2c). An RNA folding analysis by Mfold showed that the ΔG values of *cr1* and *cr2* were comparable to that of *cr* (ΔG_*cr*_ = − 56.80 kcal/mol; ΔG_*cr1*_ = − 51.50 kcal/mol; ΔG_*cr2*_ = − 52.90 kcal/mol)^26^.

### Screening for a *cis* repressor variant that endows the selection system with tightly regulated tunable gene expression

In order to optimize the *cis* repressor-equipped selection system, we took advantage of the inherent indel-prone amplification of *cr* to construct the library of *cis* repressor mutants (*cr*_*lib*_) in the pAT-*cr*_*lib*_-TEM library plasmid. We expected that some library members might exhibit less stringent repression and allow wild-type TEM to confer growth to *E. coli* XL1-Blue only at high concentrations of aTc. To screen for desired *cr* variants, *E. coli* cells were transformed with the library plasmid and plated on LB Cm30. Thirty-six transformants were picked and streaked on LB Amp50 agar plates containing 0 or 50 nM aTc. Interestingly, only one *cr* variant, designated *cr3*, was found to completely inhibit the growth of the host cells in the absence of aTc, while supplementing 50 nM aTc restored cell growth. We then carried out a growth assay of XL1-Blue/pAT-*cr3*-TEM on LB Amp50 containing varying concentrations of aTc to determine the dynamic range of P_tet_-regulated expression in the pAT-*cr3*-TEM plasmid (Fig. 2a). The colony size of the *E. coli* strain was found to increase as higher concentrations of aTc were added to LB Amp50. At 50 nM aTc, the size of XL1-Blue/pAT-*cr3*-TEM colonies was comparable to that of the *E. coli* strain that constitutively expresses TEM. DNA sequencing showed that *cr3* contained a 9-bp deletion in the region that formed an extensive mRNA hairpin structure predicted by Mfold^26^ (Fig. 2c). The more relaxed regulation of TEM expression in XL1-Blue/pAT-*cr3*-TEM was likely to derive from the shortened hairpin structure of *cr3* (ΔG_*cr3*_ = − 32.90 kcal/mol).

Western blot was performed to further examine the levels of wild-type TEM expression in pAT-TEM and pAT-*cr3*-TEM. XL1-Blue/pAT-TEM and XL1-Blue/pAT-*cr3*-TEM were grown to mid-log phase and induced with different aTc concentrations. The result showed that, in XL1-Blue/pAT-TEM, 25 nM aTc was required to induce TEM expression to a level detectable by western blot, while TEM expression in XL1-Blue/pAT-*cr3*-TEM was not observed even at 2000 nM aTc (Figure S2). In contrast to the western blot analysis, the growth assay demonstrated aTc-dependent growth phenotype in XL1-Blue/pAT-*cr3*-TEM (Fig. 2a). These data suggested that incorporating *cr3* to the P_tet_-regulated system allowed tunable gene expression at extremely low levels. We expected that the combined dynamic range of the pAT and pAT-*cr3* vectors should be useful for providing selection pressure for directed evolution of poorly active enzymes towards high catalytic activity.

### Directed evolution of the E166Y TEM mutant

As a proof of concept to validate our *cis* repressor-equipped selection system, a directed evolution experiment on a crippled mutant of TEM was performed. We reasoned that, since the wild-type TEM is a diffusion-limited enzyme, it is unlikely that further catalytic improvement can be readily achieved. To demonstrate an evolvable range of TEM in the selection system, the E166Y mutant of TEM was chosen as the evolutionary starting point. Among class A β-lactamases, which includes TEM, Glu166 is a highly conserved active site residue and serves as a general base. This catalytically crucial residue is involved in the abstraction of proton from the hydroxyl group of Ser70, a key catalytic residue responsible for the nucleophilic attack on the β-lactam ring^29–31^. Amino acid substitutions at Glu166 lead to dramatic decreases in catalytic activity, thereby reducing organismal fitness under selective environments. Even the E166D mutation, which provides the same side chain functional group, resulted in 36- and 65-fold decreases in k_cat_ and k_cat_/K_M_, respectively^32^, suggesting that TEM has been highly optimized for glutamate at this position and searches for alternative solutions in nature are impeded by the fitness constraint. For the E166Y mutant, the k_cat_ and k_cat_/K_M_ values are lowered by 8000-fold and 790-fold, respectively (Table 1). Directed evolution of the mutant would, therefore, allow a TEM sequence space in which tyrosine is used at this position to be explored. Since two nucleotide substitutions are needed for the tyrosine codons to change to the glutamate codons, random mutagenesis is unlikely to produce Y166E revertant.

**Table 1:** The kinetic parameters of TEM variants.

TEM variant	k_cat_ (s^−1^)	K_M_ (µM)	k_cat_/K_M_ (M^−1^ s^−1^)	Fold improvement	
				k_cat_	k_cat_/K_M_
Wild type	960 ± 65	250 ± 45	(3.8 ± 0.73) × 10^6^	–	–
E166Y	0.12 ± 0.006	25 ± 7.9	(4.8 ± 1.5) × 10^3^	1	1
YR1-3	0.31 ± 0.003	11 ± 0.6	(2.8 ± 0.15) × 10^4^	2.6	5.8
YR1-5	0.74 ± 0.011	15 ± 1.3	(4.9 ± 0.43) × 10^4^	6.2	10
YR2-4	1.6 ± 0.02	9.6 ± 0.9	(1.7 ± 0.17) × 10^5^	13	35
YR2-5	1.1 ± 0.02	16 ± 1.1	(6.9 ± 0.49) × 10^4^	9.2	14
YR3-3	12 ± 0.2	43 ± 2.5	(2.8 ± 0.17) × 10^5^	100	58
YR4-4	9.6 ± 0.13	15 ± 1.2	(6.4 ± 0.52) × 10^5^	80	130
YR4-9	25 ± 0.5	43 ± 2.9	(5.8 ± 0.41) × 10^5^	210	120
YR5-2	25 ± 1.0	12 ± 3.2	(2.1 ± 0.57) × 10^6^	210	440
YR5-9	22 ± 0.3	17 ± 1.3	(1.3 ± 0.10) × 10^6^	180	270

The measurements were conducted in 20 mM Tris–HCl pH 7.5 and 50 mM NaCl at 30 °C. The Michaelis–Menten plots of TEM variants are shown in Figure S3. The statistical analysis of the k_cat_ and k_cat_/K_M_ values are shown in Tables S2 and S3, respectively.

Our in vivo directed evolution workflow entails three key steps: (1) construction of random mutant libraries, (2) selection of evolved enzyme variants, and (3) verification of improved growth phenotypes. In each round of directed evolution, a library of randomized mutants was created in a selection vector by either error-prone PCR (epPCR) or DNA shuffling followed by epPCR. The pAT plasmid was used as the selection vector in initial rounds to determine the extent to which TEM variants can be evolved in the system. When pAT failed to provide enough selection pressure, we then switched to pAT-*cr3* to further evolve the catalysts. This vector exchange allowed us to determine additional selection pressure provided by pAT-*cr3*. To carry out in vivo selection, *E. coli* XL1-Blue was transformed with the library and plated on LB Amp50 containing aTc concentrations that prevent parental clones to fully grow. The size of the library can be estimated by plating the transformed cells on LB Cm30. In general, we obtained the library size of between 1.3 × 10^5^ and 1.4 × 10^7^ and selected twelve clones from selective plates that have the number of colonies corresponding to 0.05 to 1.0% survival (Table S4). To confirm the growth phenotype of the selected clones and to find suitable conditions for in vivo selection in the next round, the mutant genes were subcloned into both selection vectors and then transformed into *E. coli* cells, which were then subjected to a growth assay.

**Figure 3:**
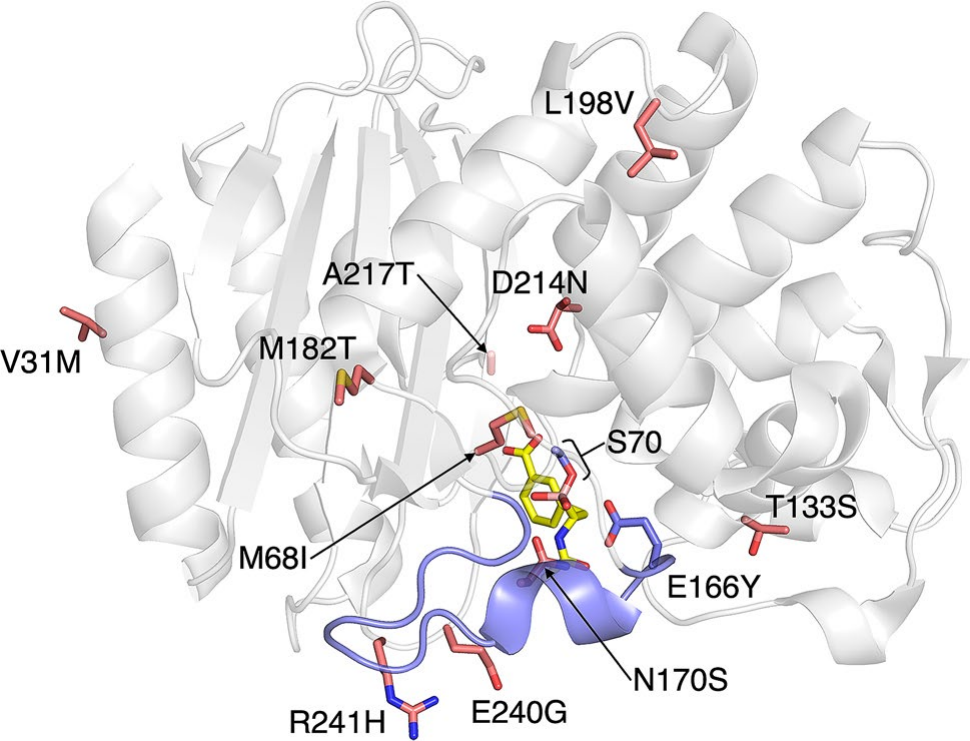
Distribution of the YR5-2 mutations in a three-dimensional structure of the wild-type TEM β-lactamase (PDB ID: 1ERM)^36^. The Glu166, Ser70, and the Ω loop are shown in purple. Mutated residues in YR5-2 are shown in pink. The boronic acid transition state analog is shown in yellow. The S4T mutation, which is located in the signal sequence, is not shown. The figure was prepared using PyMOL^37^.

In the first round of directed evolution, the E166Y TEM gene was diversified by epPCR. The first-round library was cloned into the pAT vector, which does not contain the *cr3* repressor, between the *Nde*I and *Xho*I sites. Improved clones were selected on LB Amp50 containing 25 nM aTc. Two evolved E166Y mutants, namely YR1-3 and YR1-5, were found to contain three and four additional mutations, respectively (Table 2). It is notable that three mutated residues, namely Met68, Asn170, and Asp214, are located in or close to the active site (Fig. 3). Met68 and Asp214 are residues located in loops that constitute the substrate binding pocket. Asn170, as well as Glu166, is part of a catalytically crucial structural element called Ω loop, which spans from residue 164 to 179^33,34^. Both Glu166 and Asn170 are highly conserved residues that form hydrogen bonds with the deacylating water molecule, which is crucial for the hydrolysis of the acyl-enzyme intermediate of the reaction^29^.

**Table 2:**
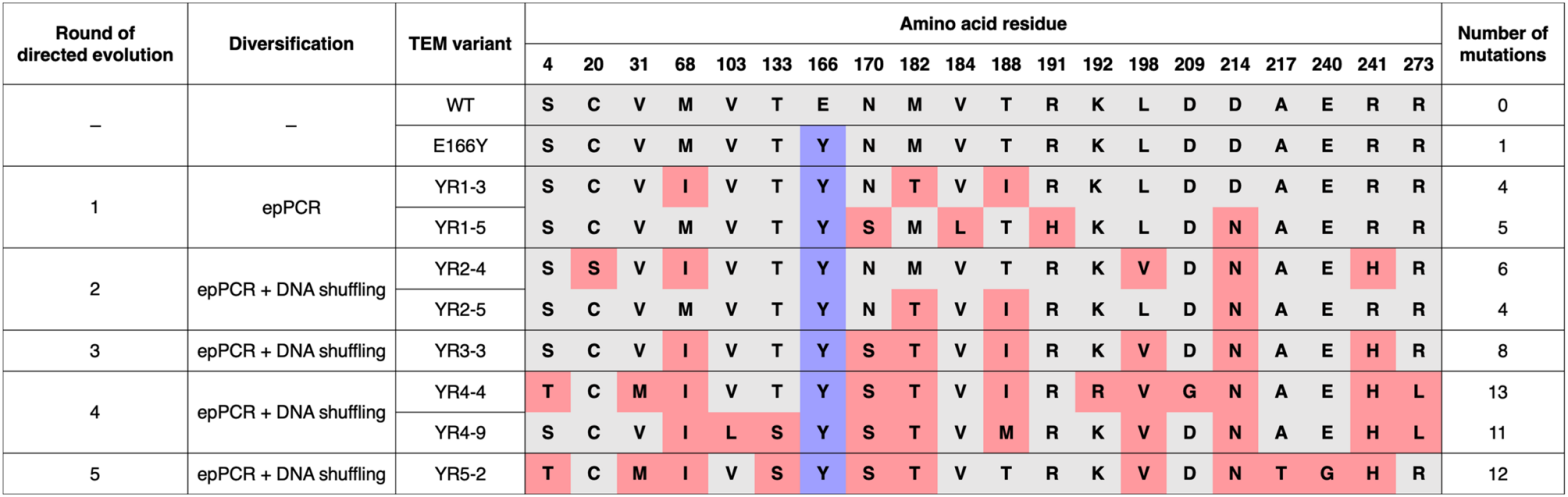
Evolutionary information of the TEM variants.

The E166Y mutation is highlighted in purple. Other mutations are highlighted in pink. Residue numbering is based on the Ambler numbering scheme^35^.

**Figure 4:**
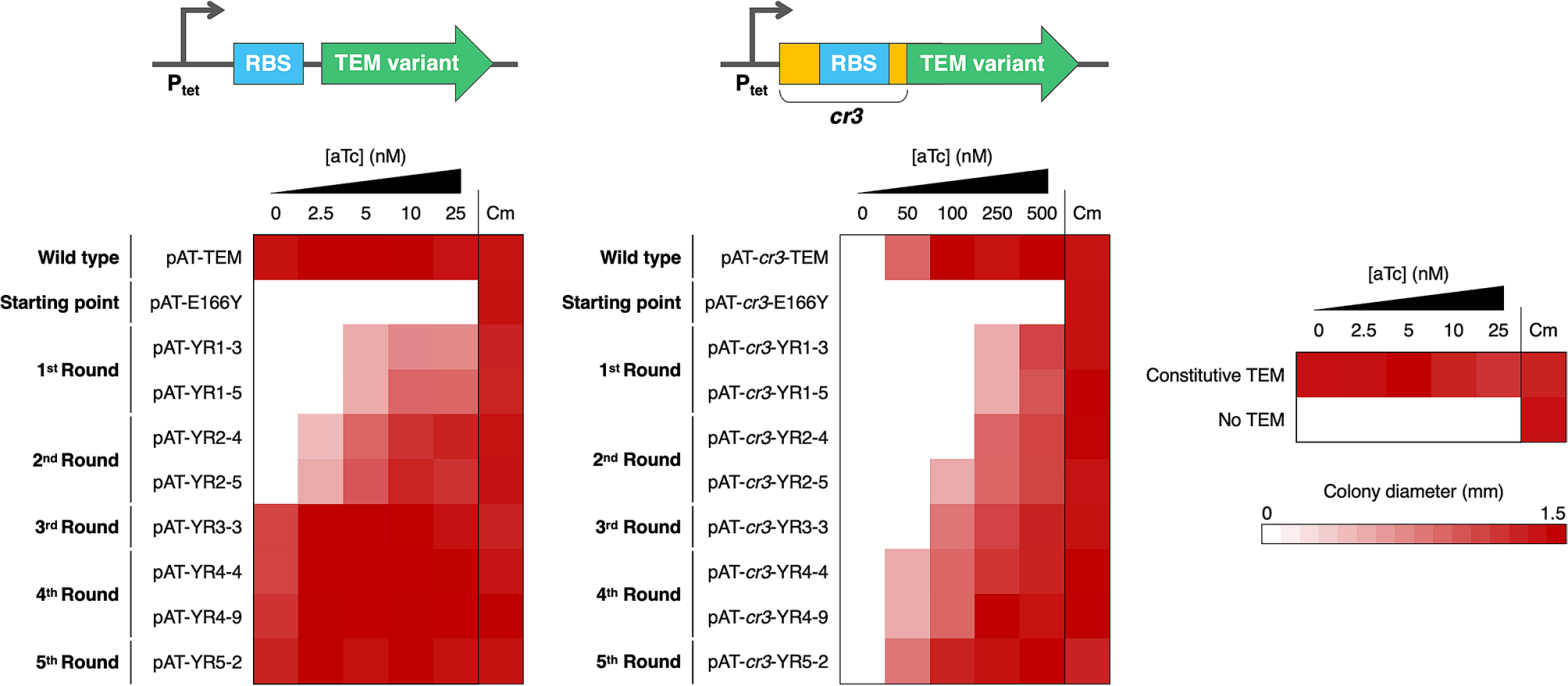
Heat map representing the average colony diameter of *E. coli* XL1-Blue expressing evolved TEM variants under the control of the pAT and pAT-*cr3* vectors. All selected gene variants were subcloned into pAT and pAT-*cr3.* Each TEM variant-expressing *E. coli* strain was grown on LB Amp50 containing varying concentrations of aTc and on LB Cm30 at 37 °C for 24 h. *E. coli* XL1-Blue containing the pAT plasmid was used as the negative control. Colony diameters were determined by ImageJ^25^. The raw data are shown in Tables S5 and S6.

From the second round onward, DNA shuffling of evolved E166Y genes from preceding rounds was performed to generate recombined mutant libraries, which were afterwards subjected to epPCR. By adding the DNA shuffling step, the libraries of enzyme variants containing various combinations of point mutations can be generated. Beneficial mutations can be combined and exhibit additive or synergistic effects, which further increase organismal fitness, while deleterious and neutral mutations that might be coselected with the beneficial mutations can be purged. The selection pressure was increased by reducing aTc concentration to 10 nM and 2.5 nM in the second and third rounds. The third round of directed evolution yielded the YR3-3 mutant, which still retains the E166Y mutation and consists of seven other mutations (Table 2). Five of which were already found in YR1-3 and YR1-5. This mutational conservation suggested that the mutations were likely to be beneficial. In contrast, two first round mutations, including V184L and R191H, were not inherited to the third round. This suggested that the mutations were either neutral or deleterious. The growth assay of XL1-Blue/pAT-YR3-3 showed that the strain was able to grow on LB Amp50 with no supplemented inducer (Fig. 4). The observed growth phenotype suggested that another selection system with higher selection pressure was required to further evolve YR3-3.

**Figure 5:**
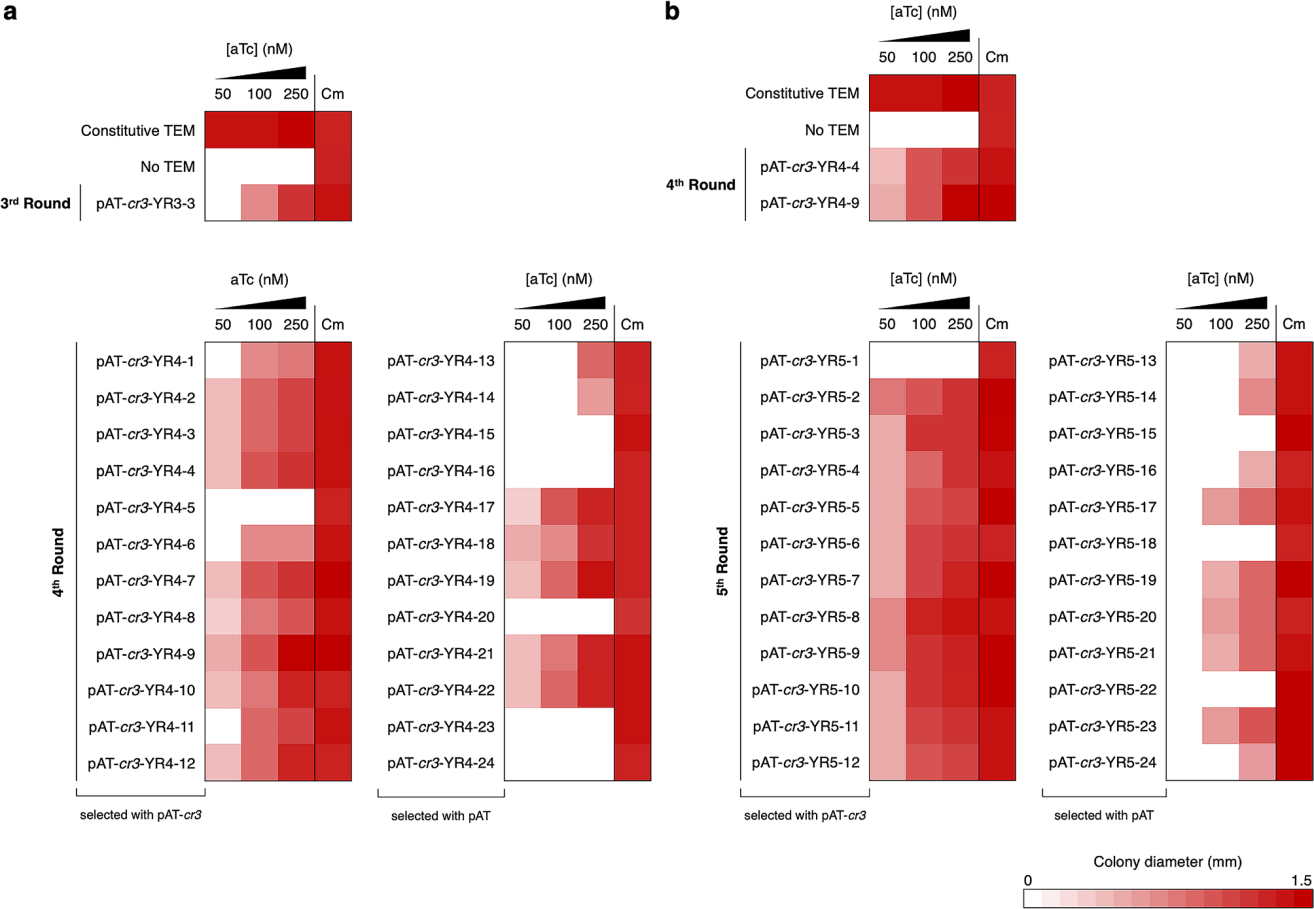
Heat map representing the average colony diameter of *E. coli* XL1-Blue expressing E166Y TEM variants selected from the fourth (**a**) and fifth round (**b**) of directed evolution. All selected gene variants were subcloned into pAT-*cr3.* Each TEM variant-expressing *E. coli* strain was grown on LB Amp50 containing varying concentrations of aTc and on LB Cm30 at 37 °C for 24 h. *E. coli* XL1-Blue containing the pAT-*cr3* plasmid was used as the negative control. Colony diameters were determined by ImageJ^25^. The raw data are shown in Tables S7 and S8.

We next utilized pAT-*cr3* instead of pAT for in vivo selection from the fourth round onward. To examine appropriate selection conditions, XL1-Blue/pAT-*cr3*-YR3-3 was grown on LB Amp50 with varying concentration of aTc. The growth assay showed that 250 nM aTc was required for normal growth and reducing aTc concentration to 50 nM completely inhibit growth (Fig. 5a), suggesting that pAT-*cr3* could provide sufficient selection pressure for the evolution of YR3-3.

In the fourth and fifth round of directed evolution, TEM mutant libraries were selected on LB Amp50 containing 100 nM and 50 nM aTc, respectively. In parallel to using pAT-*cr3*, the pAT vector was also employed to compare the application of the two systems in the evolution of highly active TEM. In the case of pAT, selections were performed using LB Amp50 with no inducer. In each round, twelve clones were picked from each library and tested for their growth characteristics under different aTc concentrations. In the fourth round, both selection systems were still able to select for evolved variants (Fig. 5a). Nevertheless, the selection using pAT-*cr3* yielded more improved clones, while growth-retarded mutants constituted the majority of clones selected with pAT. In the fifth round, evolved variants were found only when pAT-*cr3* was used as the selection plasmid, while all mutants selected with pAT showed impaired growth phenotypes, which was likely due to the introduction of deleterious mutations under neutral drift (Fig. 5b).

These two rounds of directed evolution utilizing pAT*-cr3* afforded three evolved variants, which contained seven consensus mutations (Table S9). A variant, designated YR5-2, showed slightly faster growth on LB Amp50 containing 50 nM aTc than the other two mutants (Fig. 5b). Throughout the course of YR5-2 evolution, eleven mutations were acquired (Table 2). Five of which, namely S4T, V31M, T133S, A217T, and E240G, were selected from the last two rounds. The locations of all mutated residues in YR5-2 are shown in Fig. 3. As we also attempted to carry out an additional round of directed evolution at 25 nM aTc, but no improved variants were obtained, the YR5-2 variant was chosen for further characterization.

### Characterization of TEM variants

To gain insights into how the E166Y TEM mutant evolved, we analyzed the steady state kinetics of TEM mutants that represent the evolutionary pathway of YR5-2. The kinetic analysis revealed that the k_cat_ and k_cat_/K_M_ of YR5-2 were 25 ± 1.0 s^−1^ and (2.1 ± 0.57) × 10^6^ M^−1^ s^−1^, respectively (Table 1). The k_cat_/K_M_ value was 440 fold higher than that of the E166Y mutant and was also in the same order of magnitude as the wild-type TEM. This improvement corresponds to − 3.7 kcal/mol of the free energy barrier from the free enzyme and substrate to the transition state^38^.

We also performed kinetic analysis of YR5-9, which was another improved variant selected from the fifth evolutionary round (Fig. 5b). Relative to the wild-type enzyme, YR5-9 contains thirteen mutations (Table S9). The kinetic parameters of the mutant (k_cat_ = 22 ± 0.3 s^−1^ and k_cat_/K_M_ = (1.3 ± 0.10) × 10^6^ M^−1^ s^−1^) were found to be comparable to those of YR5-2, indicating that the selection system was effective at selecting highly efficient enzyme variants.

In the first round of directed evolution, the YR1-3 and YR1-5 variants showed 5.8- and 10-fold increase in the k_cat_/K_M_ values. Four first round mutations (M68I, N170S, M182T, and D214N) were found in both YR3-3 and YR5-2. This evolutionary conservation reflects the functional significance of each mutation. We inferred the roles of mutations from previous functional and structural studies of β-lactamase mutants. As Met68 is situated on the same loop as the catalytic Ser70, M68I might be involved in positioning Ser70 residue to adapt to the bulkiness of the E166Y mutation. M182T is located at a site distal to the active site. Previous studies showed that this mutation served as a global suppressor of deleterious mutations affecting protein stability^39,40^. Asn170 in the wild-type TEM hydrogen bonds with the nucleophilic water molecule. A previous structural study of the E166Y mutant of class A β-lactamases revealed that the nucleophilic water molecule and the acyl-enzyme intermediate adopted both catalytically competent and incompetent conformations^41,42^. N170S might provide a more optimal positioning of the water molecule and/or stabilize the more productive conformation of E166Y TEM mutants. D214N is located on a loop that forms part of the active site. A previous study suggested that Asp214 is protonated and interacts with Asp233^43^. D214N might prevent destabilizing interaction caused by the aspartyl dyad when both residues are deprotonated. Intriguingly, most class A penicillinases and carbapenemases have asparagine at this position^30^.

Compared to YR2-4 and YR2-5, the YR3-3 mutant gained 7.5- and 11-fold increase in k_cat_ to 12 s^−1^. The additive effects of combining the four first round mutations mentioned above might explain this dramatic k_cat_ improvement. YR3-3 also contains three additional mutations (T188I, L198V, and R241H). Previous studies showed that these mutations acted as second site suppressors for deleterious mutations^40,44^. While T188I and L198V are surface residues located further away from the active site, R241H is situated on the β turn that interacts with the Ω loop. R241H might play a role in stabilizing catalytically productive Ω loop conformations. In the last two rounds of evolution, YR5-2 gained five more mutations (S4T, V31M, T133S, A217T, and E240G) and lost the T188I mutation. Compared to YR3-3, YR5-2 gained 7.5- and 2.1-fold improvement in k_cat_/K_M_ and k_cat_. This kinetic enhancement of the fifth-round mutant indicated that pAT-*cr3* provided sufficient selection pressure for the evolution of highly active catalysts.

**Figure 6:**
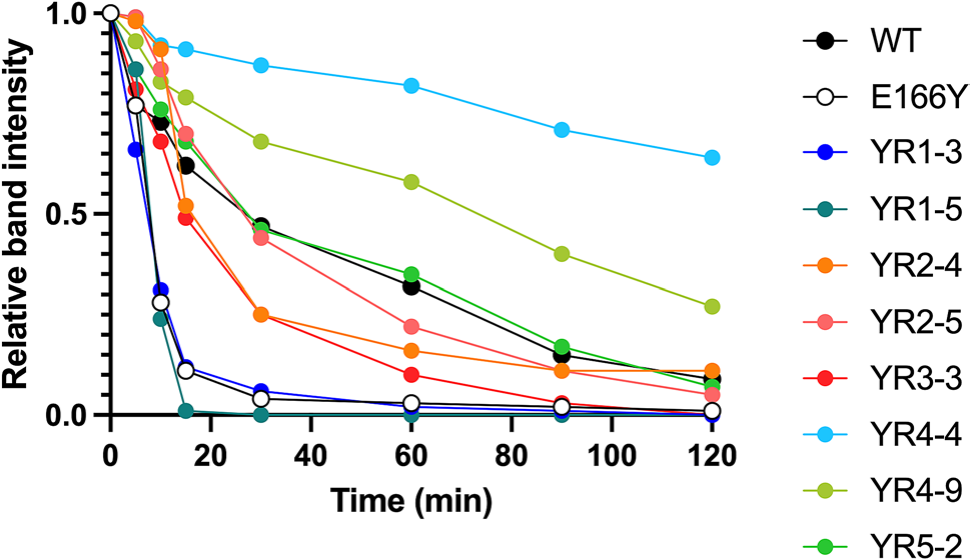
Susceptibility of TEM variants to trypsin digestion. Purified TEM variants (0.5 mg/ml) were incubated with 0.01 mg/ml trypsin in 20 mM Tris–HCl pH 7.5 and 50 mM NaCl at 25 °C. Aliquots from different time points were analyzed by SDS-PAGE. The intensity of full-length protein bands was analyzed by ImageJ^25^.

Catalyzed rates are dependent not only on the kinetic parameters but also on the concentration of enzymes. Stable proteins can persist intracellularly and extracellularly for longer periods, thereby leading to increased protein concentrations. To investigate if the improved E166Y TEM variants have also evolved for higher stability, the wild-type TEM, the E166Y TEM mutant, and its evolved variants were subjected to limited proteolysis with trypsin. Partial tryptic digestion revealed that the E166Y mutation dramatically decreased the stability of the enzyme (Fig. 6 and Figure S4). The full-length protein band of the E166Y mutant disappeared after incubating with trypsin for 30 min, while the wild-type enzyme was still visible after 120-min in the same conditions. This might be due to the more extended Ω loop conformation in the enzyme mutant^42^. Resistance against trypsin degradation was incrementally increased in evolved mutants selected in the second to the fourth round, which yielded YR4-4, the most stable mutant. Nevertheless, a significant decrease in resistance to trypsin was observed in the final round of directed evolution. Compared to YR4-4 and YR4-9, YR5-2 was found to be more susceptible to trypsin. This result suggested that the evolution of YR5-2 was subject to an activity-stability trade-off. Overall, our limited proteolysis result demonstrated that the acquired mutations contributed to not only enhanced kinetic parameters but also to the stability of the evolved TEM mutants. Despite a longstanding notion that enzyme evolution is constrained by activity-stability trade-offs, our findings showed that catalytic activity and stability could be simultaneously optimized during evolution.

## Discussion

Directed evolution has been a widely used strategy for enzyme engineering^1,45,46^. Adaptive evolution algorithm of directed evolution can be achieved by repeating the process of (1) gene diversification to create a pool of randomized mutants, (2) selection or screening of improved enzyme variants, and (3) amplification of the genes of the selected/screened variants for the next round of directed evolution. It is estimated that ~ 30–50% of random mutations are deleterious, while ~ 50–70% are neutral. Only a small fraction (~ 0.01–0.5%) of the mutations is beneficial^47^. Mutating key catalytic residues often leads to significant or complete loss of activities, while substituting buried residues can destabilize proteins and hamper their functions. Thus, the ability to differentiate improved protein variants from those with unaltered or retarded activities is critically important for directed evolution. By coupling the growth of an organism to the activity of the protein of interest, in vivo selection systems offer a simple and robust approach that can be implemented as part of directed evolution workflows. The selection pressure of the systems can be adjusted such that only improved mutants can survive and grow. Since the rate of an enzyme-catalyzed reaction is dependent on the kinetic parameters and enzyme concentration, restricting the expression levels of the target enzymes poses selection pressure on the pool of randomized enzyme mutants. Those that can overcome the metabolic bottleneck by acquiring beneficial mutations that enhance their kinetics and stability are selected for the next round of directed evolution.

In this study, we devised an in vivo selection system, in which a translationally suppressing *cis* repressor and P_tet_ were incorporated to impart tight and tunable selection pressure. By using the wild-type TEM as the model enzyme, we found that, without the *cis* repressor, the selection system failed to provide high selection pressure for the evolution of highly active enzyme variants. Despite the fact that a number of previous studies have shown that P_tet_ exhibits low basal level of gene expression^48,49^, our results revealed that that level of uninduced expression was high enough for the wild-type TEM to allow the *E. coli* host cells to be fully resistant to ampicillin. This could be because the wild-type TEM is a highly efficient enzyme, whose catalytic activity is partially diffusion-limited^23^. Even low level of TEM expression is sufficient for degrading the antibiotic. To solve this problem, we screened a library of *cis* repressors with variable length for one that could shift the dynamic range of P_tet_ towards a lower expression range. We found a *cis* repressor-equipped expression plasmid that enabled tightly regulated expression of the wild-type TEM. The *E. coli* host cells containing the plasmid exhibited aTc-dependent growth without expression leakage in the absence of aTc. This result has an implication in using RNA-based genetic parts to construct regulatory devices. Unlike protein-based counterparts, RNA-based regulators are more easily designed and engineered due to their predictable RNA secondary structures and well-developed models for RNA thermodynamics^50,51^. In our work, initial *cis* repressor sequences were found to be too stringent. The screening of *cis* repressor library allowed us to tune expression suppression to a level suitable for directed evolution of highly active TEM.

Our directed evolution experiment using the E166Y TEM mutant as the starting point showcases the evolvable range of the enzyme in the in vivo selection systems. E166Y is a crippling active site mutation that destabilizes the protein and reduces the k_cat_/K_M_ value by 790 folds. The mutated residue plays a key role in the acylation-deacylation cycles of the reaction^29,31^. Five evolutionary rounds, while still retaining the active site mutation, not only restored k_cat_/K_M_ but also improved the stability of the enzyme to a level comparable to the wild-type TEM. Even though the *cis* repressor-equipped system was exploited for the last two rounds of directed evolution, the growth of the E166Y mutant and its evolved variants under the *cis* repressor-equipped selection system suggests that the system could be used throughout the entire evolutionary experiment.

With regards to the evolution of kinetic parameters, the kinetics of the TEM variants is exemplary of in vivo selection under the Michaelis–Menten regime in which the growth of host cells correlates with the rates of a catalyzed reaction. Since the ampicillin concentration of ~ 135 μM was used and the K_M_ values of the E166Y mutant and almost all selected variants are much less than the substrate concentration, the rate of ampicillin hydrolysis was largely dependent on k_cat_. Under selective conditions in which K_M_ ≪ [S], enzyme variants with higher k_cat_ and stability can confer higher growth rate to the host cells. The libraries of randomized TEM variants were, therefore, under the selective pressure for improving their k_cat_ values and stability. Overall, the 210-fold improvement in k_cat_ was the primary contributing factor for the increase in k_cat_/K_M_. Although the wild-type TEM and YR5-2 have comparable k_cat_/K_M_ values, the observed growth conferred by both variants was found to be significantly different. Again, this growth discrepancy can be explained by the 38-fold higher k_cat_ of the wild-type TEM. In some cases, the improved growth phenotypes cannot be explained by an increase in k_cat_. For example, the k_cat_ value of YR4-4, a fourth-round mutant, was actually found to be less than that of YR3-3, a third-round mutant. Limited proteolysis revealed that YR4-4 was more resistant to tryptic digestion than YR3-3. This higher stability is an indicator of improved protein folding of the evolved mutant.

Since higher kinetics and stability are desirable attributes of enzymes exploited for biotechnological applications, our *cis* repressor-equipped selection system should be useful for engineering a wide range of enzymes to meet these industrial requirements. Similar RNA-based strategy for tuning selection pressure can be conveniently done in industrially relevant hosts to allow directed evolution in the genetic and environmental contexts of the organisms. Besides the use of the selection system for biotechnological applications, we also envision its application in answering many basic evolutionary questions. One of which is why most enzymes have not been evolved to catalyze their chemical reactions at the diffusion limit. Is it because there has been no selection pressure for higher catalytic activity or because their inherent reaction mechanisms pose another limit to enzyme evolution?

## Methods

### General DNA manipulation

DNA was manipulated according to the standard protocols with some modifications according to the instructions of reagent manufacturers. Restriction enzymes, T4 DNA ligase, Phusion DNA polymerase, and DNase I were purchased from New England Biolabs. DNA purification was carried out with DNA Clean & Concentrator Kit (Zymo Research). To purify DNA from excised agarose gel, Zymoclean Gel DNA Recovery Kit (Zymo Research) was used. Plasmid DNA isolation was carried out with ZymoPURE Plasmid Miniprep Kit (Zymo Research). Transformation of *E. coli* XL1-Blue chemically competent cells was carried out by an optimized heat shock method^52^.

### PCR conditions

For general DNA cloning, all PCR reactions were carried out in 50 μl of reaction mixture, which contained 1 U of Phusion DNA polymerase, 0.5 μM of each primer, 200 μM of dNTPs, appropriate amount of DNA template, and 1X Phusion HF Buffer. PCR was performed with initial denaturation at 98 °C for 30 s and continued with 30 amplification cycles including a denaturation step at 98 °C for 5 s, an annealing step at a primer-specific temperature for 10 s, and an extension step at 72 °C for 45 s. The reaction was finally incubated at 72 °C for 5 min. The list of all primers and their annealing temperature is shown in Table S10.

### Plasmid construction

To construct the pAT plasmid, pKTR-mMjCMneg was amplified with PacI-KTR-F and AvrII-KTR-R to generate a 2144-bp plasmid fragment lacking the TEM gene and its native promoter. A chloramphenicol resistance gene was amplified from pGro7 (Takara Bio) with AvrII-Cm-F and PacI-Cm-R and subsequently ligated with the plasmid fragment via the *Avr*II and *Pac*I sites to create pAT-mMjCMneg. A 1298-bp stuffer DNA fragment was digested from pKTCTET-0 with *Nde*I and *Xho*I, and inserted into pAT-mMjCMneg via the same sites to generate the pAT vector. The pAT-TEM plasmid was constructed by amplifying the wild-type TEM gene from pKTR-mMjCMneg with the primers NdeI-Amp-F and XhoI-Amp-R and inserting into pAT between the *Nde*I and *Xho*I sites. The resulting plasmid was named pAT-TEM.To incorporate the *cr* sequence into pAT-TEM, a synthetic *cr* fragment was amplified with the primers HindIII-tetR-F and NdeI-cis-R to produce an 845-bp fragment containing *cr*. A 2614-bp pAT-TEM fragment was amplified with the primers NdeI-Amp-F and HindIII-pAT-R. The pAT-TEM fragment and the *cr* fragment were ligated together via the *Hind*III and *Nde*I sites to generate the pAT-*cr*-TEM plasmid. To construct a constitutive expression vector for the wild-type TEM, the wild-type TEM gene and its native constitutive promoter were amplified from pKTR-mMJCMneg with the primers EcoRI-pAmp-F and XhoI-Amp-R and cloned into pAT-*cr*-TEM via the *EcoR*I and *Xho*I sites. The resulting plasmid was designated pAT-TEM_c_. DNA sequencing was performed to verify the DNA sequences of all constructed plasmids.

### Site-directed mutagenesis

The E166Y mutant of TEM was constructed by overlap extension PCR. The wild-type TEM gene was amplified by PCR with two pairs of primers to generate 5′ and 3′ gene fragments. The 5′ fragment was amplified with the primers NdeI-Amp-F and E166Y-R. The 3′ fragment of the mutant gene was amplified with the primers E166Y-F and XhoI-Amp-R. The 5′ and the 3′ fragments were then combined, and three cycles of overlap extension was done with initial denaturation at 98 °C for 30 s and continued with 30 amplification cycles including a denaturation step at 98 °C for 5 s, an annealing step at 52 °C for 10 s, and an extension step at 72 °C for 10 s. After adding the primers NdeI-Amp-F and the XhoI-Amp-R to the overlap extension reaction, the full-length mutant gene was amplified by 30 cycles of PCR using the same program except that the annealing temperature was set to 59 °C. The amplified TEM mutant gene was ligated with pAT and pAT-*cr3* via the *Nde*I and *Xho*I sites to create pAT-E166Y and pAT-*cr3*-E166Y, respectively. The DNA sequence of the E166Y TEM gene was confirmed by DNA sequencing.

### Growth assay

The complementation ability of TEM variants on the growth of *E. coli* XL1-Blue on LB Amp50 was assessed in triplicate. *E. coli* XL1-Blue expressing a TEM variant was grown in LB Cm30 broth overnight at 37 °C. *E. coli* XL1-Blue pAT or pAT-*cr3*, which contains no TEM gene, was used as a negative control. *E. coli* XL1-Blue pAT-TEM_c_, which constitutively expresses TEM, was used as a positive control. The overnight cultures were diluted to the OD_600_ of 1 and then for another 10^5^ fold. Diluted cells were spread on LB Amp50 agar plates containing different concentrations of aTc and on an LB Cm30 agar plates. After incubating the plates at 37 °C for 24 h, the photos of the plates were taken with iPhone 11 Pro Max using the automatic exposure mode and analyzed by ImageJ^25^. The diameters of all colonies on a plate were determined by using a 6-mm filter paper disc as the standard.

### Western blot analysis

*E. coli* XL1-Blue strains containing pAT-TEM or pAT-*cr3*-TEM were grown in LB Cm30 broth overnight at 37 °C. 100 μl of the overnight cultures was used to inoculate 5 ml LB broth. When the OD_600_ reached ~ 0.5, aTc was added at different final concentrations (0, 5, 10, and 25 nM). The cultures were further shaken at 200 rpm at 37 °C for another 6 h and diluted to the OD_600_ of 2. Cells were harvested by centrifugation, disrupted by sonication in 20 mM Tris–HCl pH 7.5 and 50 mM NaCl, and centrifuged again to remove insoluble cell debris. The supernatants were run on SDS-PAGE. The proteins were transferred from the polyacrylamide gel to a PVDF membrane with Mini Trans-Blot Electrophoretic Transfer Cell (Bio-Rad). The membrane was incubated at room temperature for 1 h in blocking solution (20 mM Tris–HCl pH 7.5, 150 mM NaCl, 0.1% Tween 20, and 5% (w/v) milk) and then in blocking solution containing His-Tag (27E8) Mouse mAb (HRP-conjugate) (Cell Signaling Technology) at 4 °C overnight. After washing with TBST buffer (20 mM Tris–HCl pH 7.5, 150 mM NaCl, and 0.1% Tween 20), the membrane was incubated with Clarity Western ECL Substrate (Bio-Rad) for 5 min. Chemiluminescent signal was visualized with ImageQuant LAS 500 (Cytiva).

### Construction of random mutant libraries

The first library was constructed by carrying out epPCR of the E166Y TEM gene. A 50-µl epPCR reaction mixture was set up by adding 1.25 U of Mutazyme II DNA polymerase (Agilent), 1.0 μM of the T7 promoter primer, 10 mM dNTPs, 10 ng of plasmid DNA template, and 1X Mutazyme II reaction buffer. The following temperature-controlled program was used for epPCR: an initial denaturation step (95 °C for 2 min), thirty amplification cycles (denaturation at 95 °C for 30 s, annealing at 45 °C for 30 s, and extension at 72 °C for 90 s) and a final extension step (72 °C for 5 min). The epPCR product was ligated with pAT via the *Nde*I and *Xho*I sites to create the first library.

For the second and third rounds of directed evolution, evolved TEM variants selected from a preceding evolutionary round were subjected to DNA shuffling followed by epPCR. To perform DNA shuffling, the variant genes were separately amplified with the T7 promoter primer and pooled equimolarly. 4.5 µg of the DNA pool was incubated on ice with 0.2 U of DNase I in the total reaction volume of 60 µl until DNA was digested to the size of ~ 100 bp. In order to synthesize a full-length gene library, 50 µl PCR reaction mixture was prepared by adding 200 ng of the digested DNA, 1 U of Phusion DNA polymerase, 200 µM of dNTPs, and 1X HF buffer. The reaction was done with initial denaturation at 98 °C for 30 s, and followed by 10 cycles of denaturation at 98 °C for 10 s, annealing at 45 °C for 10 s, and extension at 72 °C for 30 s. Subsequently, 1.0 µM of T7 promoter primer was added to the reaction mixture. PCR was performed for another 20 cycles using the same program. The PCR product was further amplified by epPCR as described for the first library. To construct the second and third libraries, the epPCR product of each round was then inserted into pAT at the *Nde*I and *Xho*I sites.

For the fourth and fifth libraries, sequence diversification was performed as outlined for the second and third libraries, except for a few differences. First, the primers Cis-F and AvrII-KTR-R were used for DNA shuffling and epPCR. The annealing temperature for DNA shuffling and epPCR was changed to 60 °C. In the final step, the epPCR products were cloned into pAT-*cr3* to generate the libraries.

### In vivo selection

100–200 ng of the TEM mutant libraries was used to transform *E. coli* XL1-Blue electrocompetent cells with MicroPulser Electroporator (Bio-Rad) according to the manufacturer’s protocol. The transformants were spread on LB Amp50 containing appropriate aTc concentrations for in vivo selection and on LB Cm30 to determine the size of the libraries. After 18 h incubation at 37 °C, 12 colonies that grew on the aTc-containing LB Amp50 were picked and streaked onto the same media along with their parental clones to compare the growth characteristics. Plasmids were then isolated from the selected clones and the TEM variant genes were subcloned into the pAT and pAT-*cr3* plasmids for the growth assay to confirm the growth phenotype of the selected TEM variants. DNA sequencing was carried out to identify mutations in the variant genes.

### Protein production and purification

Genes encoding TEM variants without their signal sequences were PCR amplified with the primers pET-F and XhoI-Amp-R. For mutants containing the V31M mutation, the primer pET-V31M-F was used instead of pET-F. 808-bp amplified gene fragments were then cloned into the pET-29b vector between the *Nde*I and *Xho*I sites to generate TEM overexpression vectors. *E. coli* BL21(DE3) containing each TEM variant-expressing pET-29b plasmid was grown overnight in 5 ml LB broth containing 50 µg/ml kanamycin (LB Kan50) at 37 °C. 250 ml LB Kan50 was inoculated with the overnight culture and shaken at 200 rpm at 37 °C. When the OD_600_ of the culture was ~ 0.5, isopropyl β-D-1-thiogalactopyranoside (IPTG) was added to the final concentration of 0.25 mM to induce protein production. After 18 h at 23 °C, cells were collected by centrifugation and disrupted by sonication in 20 mM Tris–HCl pH 7.5, 50 mM NaCl, and 20 mM imidazole. Insoluble cell debris was subsequently removed by centrifugation. Cleared cell lysates were loaded into Ni Sepharose 6 Fast Flow (GE Healthcare) columns equilibrated with 20 mM Tris–HCl pH 7.5, 50 mM NaCl, and 20 mM imidazole. The columns were washed three times with the same buffer. Proteins were eluted from the columns with 20 mM Tris–HCl pH 7.5, 50 mM NaCl, and 200 mM imidazole. Eluted proteins were concentrated and buffer exchanged into 20 mM Tris–HCl pH 7.5 and 50 mM NaCl by using centrifugal concentrator filters with 10-kD molecular weight cut-off. The purity of the purified proteins was examined by SDS-PAGE. Protein concentration was determined by measuring the absorbance at 280 nm.

### Steady-state kinetic analysis

The kinetic assays of TEM variants were carried out in triplicate according to a method described in a previous study^53^. Reactions were set up with different concentrations of ampicillin in 20 mM Tris–HCl pH 7.5 and 50 mM NaCl at 30 °C. Depending on the catalytic activity of each TEM variant, purified enzymes were added at final concentrations ranging from 0.005 to 1.0 μM. The hydrolysis of ampicillin was spectroscopically monitored at 235 nm with Shimadzu UV-2600 UV–VIS Spectrophotometer. Decreases in absorbance at 235 nm within the first 10% of the reaction were converted to initial rates by using the change in molar extinction coefficient value (∆ɛ) of − 900 M^−1^ cm^−1^. The initial rates obtained with different concentrations of ampicillin were fitted to the Michaelis–Menten equation by Prism 9 software. The kinetic parameter data were subjected to multiple t-test analysis using Prism 9 software.

### Limited proteolysis

Limited proteolysis was performed by incubating 0.5 mg/ml purified TEM variant with 0.01 mg/ml trypsin in 20 mM Tris–HCl pH 7.5 and 50 mM NaCl at 25 °C. Aliquots of each reaction mixture was collected at particular time points and mixed with the SDS-PAGE loading buffer (62.5 mM Tris–HCl pH 6.8, 2% (w/v) SDS, 10% glycerol, and 0.01% (w/v) bromophenol blue) to stop the reaction. Quenched samples were run on SDS-PAGE. The intensity of full-length protein bands was analyzed by ImageJ^25^.

## Supplementary information

Supplementary Information.
