# Supplementary material for: An in vivo selection system with tightly regulated gene expression enables directed evolution of highly efficient enzymes
Source: Sci Rep. 2021 Jun 3;11:11669. doi: 10.1038/s41598-021-91204-4 (PMC8175713; doi:10.1038/s41598-021-91204-4)
Supplement: Supplementary file 1 — Supplementary Information. [file 41598_2021_91204_MOESM1_ESM.pdf]

Supplementary data

An *in vivo* selection system with tightly regulated gene expression enables directed evolution of highly efficient enzymes

Parinthon Nearmnala[1], Manutsawee Thanaburakorn[1], Watanalai Panbangred[1,2], Pimchai Chaiyen[3] and Narupat Hongdilokkul[1,2,*]

1 Department of Biotechnology, Faculty of Science, Mahidol University, Bangkok 10400, Thailand

2 Mahidol University and Osaka University Collaborative Research Center for Bioscience and Biotechnology, Faculty of Science, Mahidol University, Bangkok 10400, Thailand

3 School of Biomolecular Science and Engineering, Vidyasirimedhi Institute of Science and Technology (VISTEC), Rayong 21210, Thailand

* Corresponding author. Email: narupat.hon@mahidol.edu

```
cr        AGTGTGATTA CATTTTCTTG TCTCAAACAA TAAGCTAGCA TTCGAGCCTC 50
Clone 1   AGTGTGATTA CATTTTCTTG TCTCAAACAA TAAGCTAGCA TTCGAGCCTC 50
Colne 2   AGTGTGATTA CATTTTCTTG TCTCAAACAA TAAGCTAGCA TTCGAGCCTC 50
Colne 3   AGTGTGATTA CATTTTCTTG TCTCAAACAA TAAGCTAGCA TTCGAGCCTC 50
Colne 4   AGTGTGATTA CATTTTCTTG TCTCAAACAA TAAGCTAGCA TTCGAGCCTC 50
Colne 5   AGTGTGATTA CATTTTCTTG TCTCAAACAA TAAGCTAGCA TTCGAGCCTC 50
Colne 6   AGTGTGATTA CATTTTCTTG TCTCAAACAA TAAGCTAGCA TTCGAGCCTC 50
Clone 7   AGTGTGATTA CATTTTCTTG TCTCAAACAA TAAGCTAGCA TTCGAGCCTC 50
Clone 8   AGTGTGATTA CATTTTCTTG TCTCAAACAA TAAGCTAGCA TTCGAGCCTC 50

cr        TCCTTCTATC GGCGTGTGAC GAGAAATCGT AATGCGTCGA TAGAAGGAGA 100
Clone 1   TCCTTCT-TC GGCGTGTGAC GAGAAATCGT AATGCGTCGA TAGAAGGAGA 99
Clone 2   TCCTTCT-TC GGCGTGTGAC GAGAAATCGT AATGCGTCGA TAGAAGGAGA 99
Clone 3   TCCTTC--TC GGCGTGTGAC GAGAAATCGT AATGCGTCGA TAGAAGGAGA 98
Clone 4   TCCTTCT--- ------TGAC GAGAAATCGT AATGCGTCGA TAGAAGGAGA 91
Clone 5   TCCTTCT--- ------TGAC GAGAAATCGT AATGCGTCGA TAGAAGGAGA 91
Clone 6   TCCTTCT--- ------TGAC GAGAAATCGT AATGCGTCGA TAGAAGGAGA 91
Clone 7   TCCTTCT--- ------TGAC GAGAAATCGT AATGCGTCGA TAGAAGGAGA 91
Clone 8   TCCTTCT--- ------TGAC GAGAAATCGT AATGCGTCGA TAGAAGGAGA 91

cr        GGTTCGA--- ---------- ---------- ---------- -CATATG    113
Clone 1   GGTTCGA--- ---------- ---------- ---------- -CATATG    112
Clone 2   GGTTCGACA- ---------- ---------- --------CC GCATATG    117
Clone 3   GGTTCGA--- ---------- ---------- ---------- -CATATG    111
Clone 4   GGTTCCGC-- -----ATGCG TCGATAGAAG GAGAGGTTCC GCATATG    131
Clone 5   GGTTCCGC-- -------ACG TCGATAGAAG GAGAGGTTCC GCATATG    129
Clone 6   GGTTCCGCAA TCGTAATGCG TCGATAGAAG GAGAGGTTCC TCATATG    138
Clone 7   GGTTCCGCA- --GTAATGCG TCGATAGAAG GAGAGGTTCC GCATATG    135
Clone 8   GGTTCCGC-- -----ATGCG TCGATAGAAG GAGAGGTTCC GCATATG    131
```

Figure S1 Multiple sequence alignment of *cr* and its mutants (Clone 1 to 8). The mutants were the result of unexpected indel-prone cloning of *cr* into pAT-TEM. The ribosome binding sites and the start codons of the gene downstream are highlighted in blue and green, respectively.

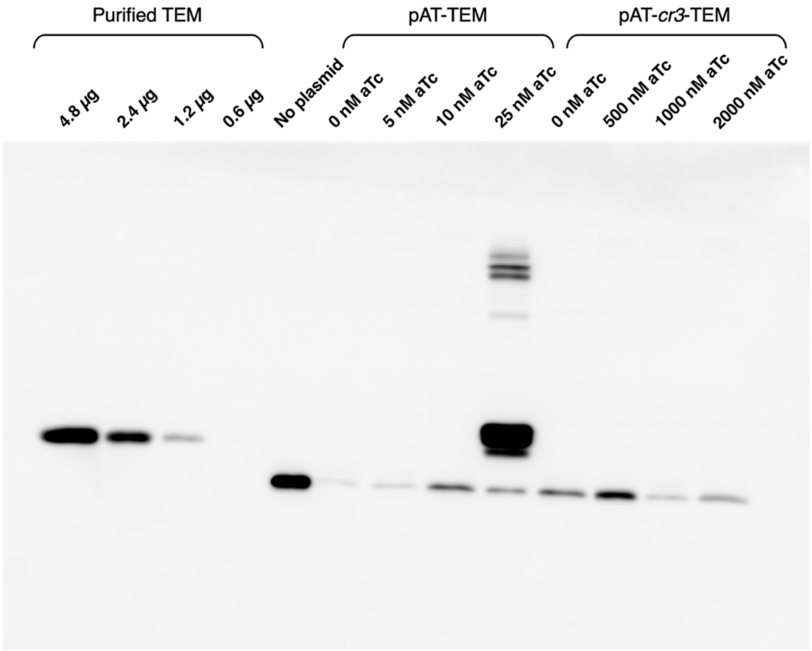

Figure S2 Western blot analysis of wild-type TEM β-lactamase expression with pAT-TEM and pAT-*cr3*-TEM. XL1-Blue/pAT-TEM and XL1-Blue/pAT-*cr3*-TEM were grown to a mid-log phase before adding aTc to the cultures at different final concentrations. Purified TEM protein was used as the control.

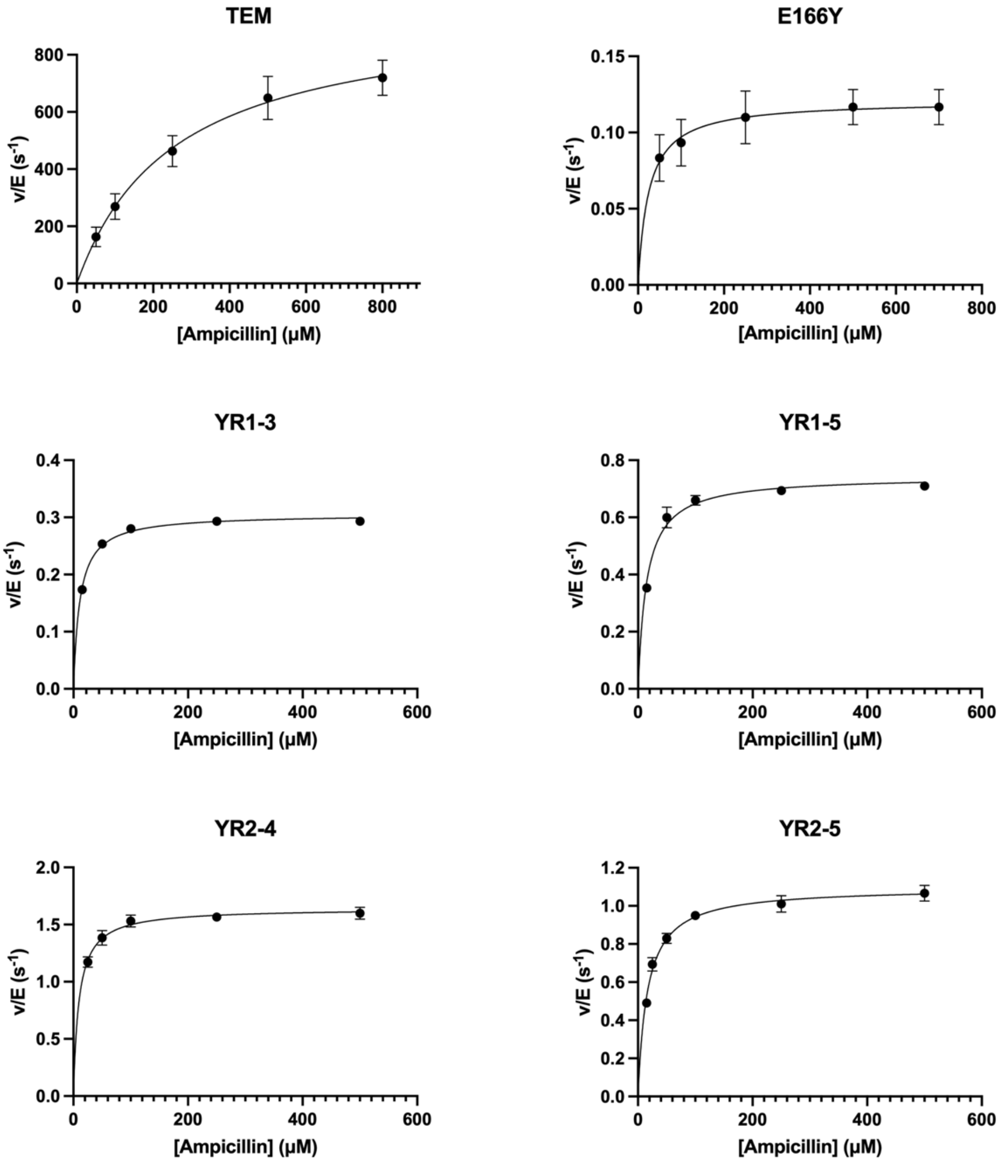

Figure S3 Michaelis-Menten plots of TEM variants. The measurements were conducted in 20 mM Tris-HCl pH 7.5 and 50 mM NaCl at 30°C.

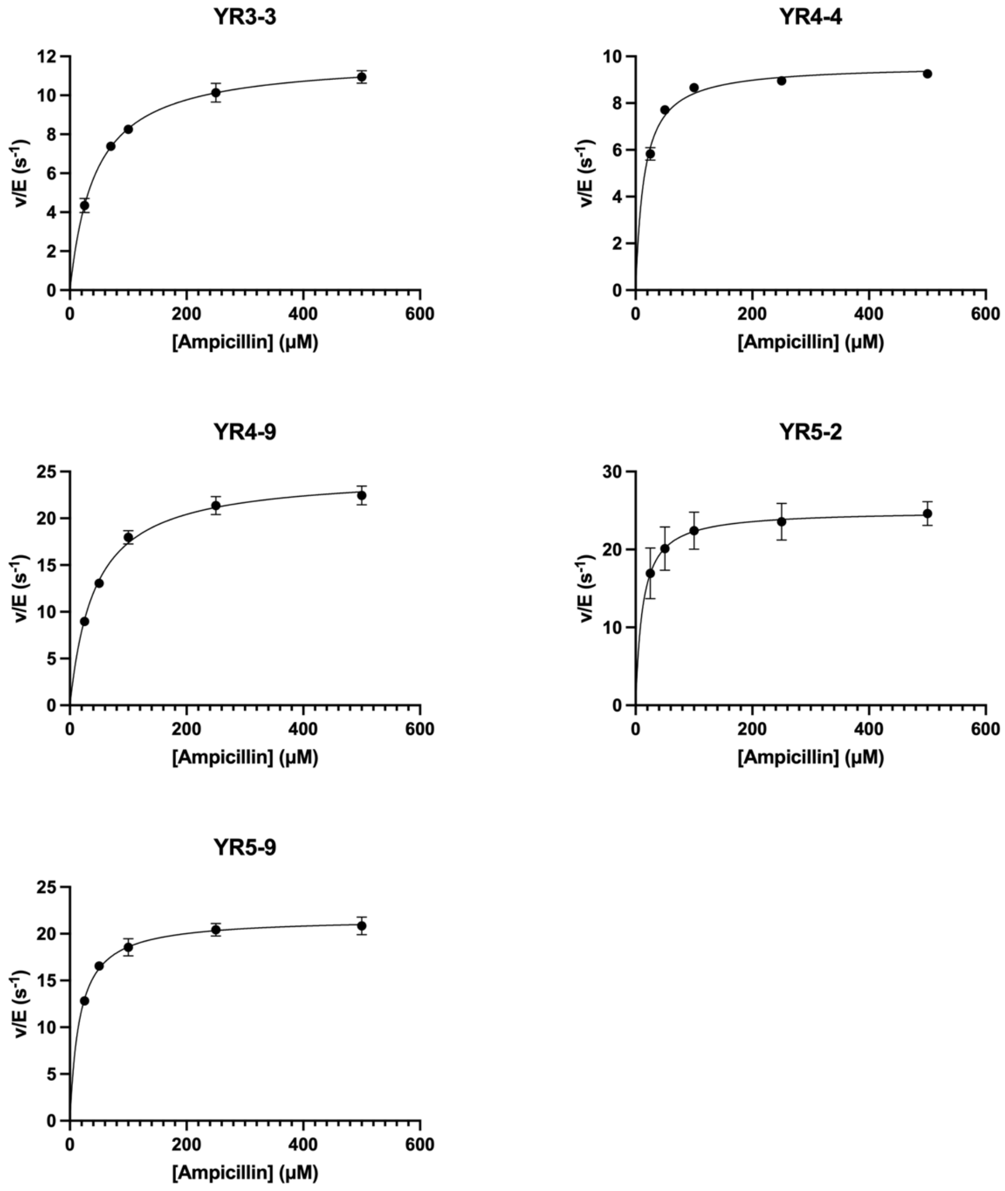

Figure S3 (continued) Michaelis-Menten plots of TEM variants. The measurements were conducted in 20 mM Tris-HCl pH 7.5 and 50 mM NaCl at 30°C.

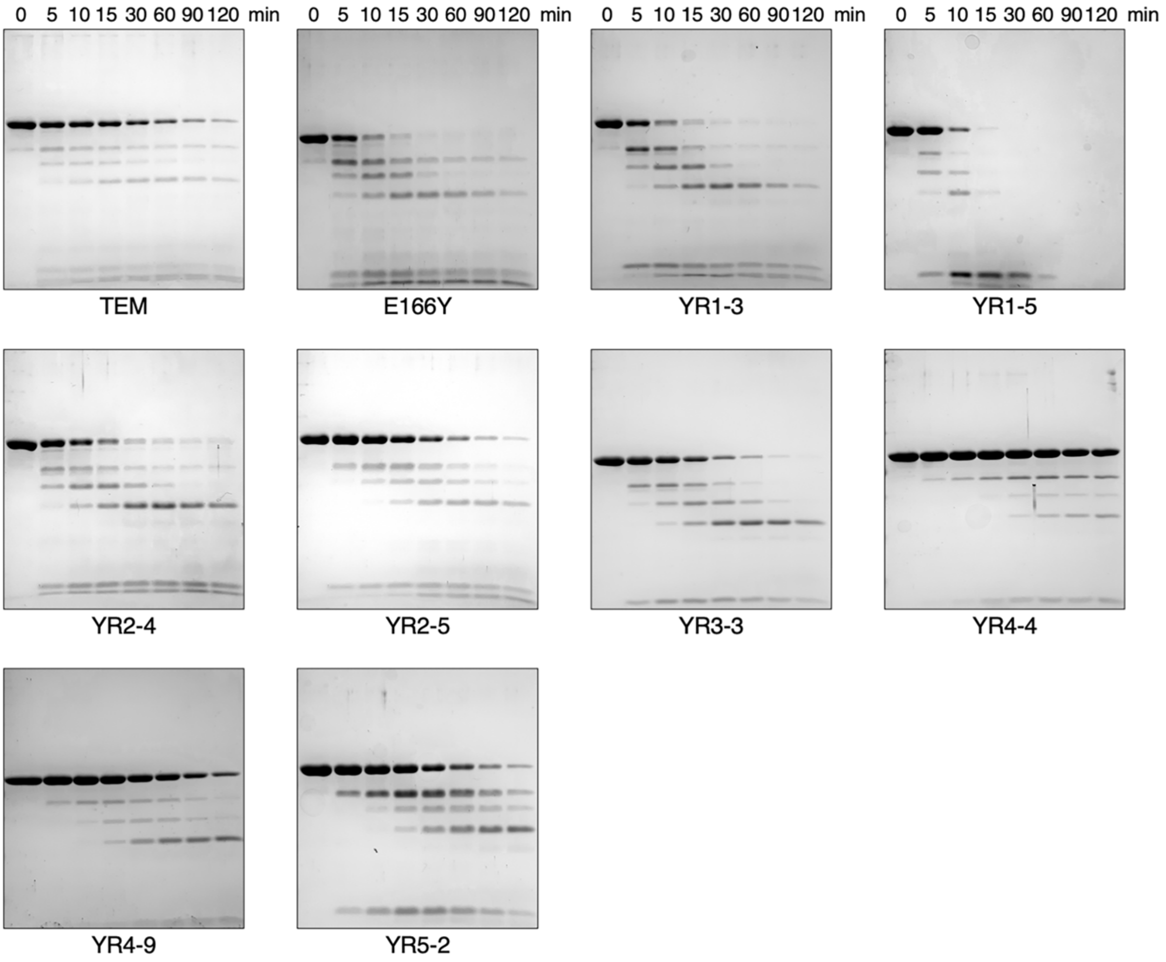

Figure S4 Susceptibility of TEM variants to trypsin digestion. Purified TEM variants (0.5 mg/ml) were incubated with 0.01 mg/ml trypsin in 20 mM Tris-HCl pH 7.5 and 50 mM NaCl at 25°C. Aliquots from different time points were analyzed by SDS-PAGE.

Table S1 Average colony diameter of *E. coli* XL1-Blue expressing the wild-type TEM β-lactamase under the control of different expression vectors. Each TEM variant-expressing *E. coli* strain was grown on LB Amp50 containing varying concentrations of aTc and on LB Cm30 at 37°C for 24 h. Colony diameters were determined by ImageJ[25].

TEM variant	Average colony diameter (mm)						
	aTc concentration (nM)						
	0	10	15	20	25	50	Cm
**Constitutive TEM**	1.6 ± 0.1	1.7 ± 0.2	1.8 ± 0.06	1.7 ± 0.06	1.8 ± 0.2	1.8 ± 0.1	1.7 ± 0.06
**No TEM**	0.0 ± 0.0	0.0 ± 0.0	0.0 ± 0.0	0.0 ± 0.0	0.0 ± 0.0	0.0 ± 0.0	1.4 ± 0.06
**pAT-TEM**	1.8 ± 0.1	1.7 ± 0.2	1.7 ± 0.0	1.7 ± 0.2	1.2 ± 0.1	0.0 ± 0.0	1.7 ± 0.06
**pAT-*cr1*-TEM**	0.0 ± 0.0	0.0 ± 0.0	0.0 ± 0.0	0.0 ± 0.0	0.0 ± 0.0	0.0 ± 0.0	1.4 ± 0.0
**pAT-*cr2*-TEM**	0.0 ± 0.0	0.0 ± 0.0	0.0 ± 0.0	0.0 ± 0.0	0.0 ± 0.0	0.0 ± 0.0	1.2 ± 0.0
**pAT-*cr3*-TEM**	0.0 ± 0.0	0.7 ± 0.0	0.9 ± 0.06	1.2 ± 0.06	1.4 ± 0.06	1.6 ± 0.3	1.4 ± 0.06

Table S2 Multiple t-test analysis on the k_{cat} values of TEM variants. The adjusted p-values are shown. The value shown in red indicates the p-value larger than 0.05.

	TEM	E166Y	YR1-3	YR1-5	YR2-4	YR2-5	YR3-3	YR4-4	YR4-9	YR5-2
TEM	-	-	-	-	-	-	-	-	-	-
E166Y	0.000208	-	-	-	-	-	-	-	-	-
YR1-3	0.000208	0.000027	-	-	-	-	-	-	-	-
YR1-5	0.000208	0.000004	0.000002	-	-	-	-	-	-	-
YR2-4	0.000208	0.000001	0.000009	0.000010	-	-	-	-	-	-
YR2-5	0.000208	0.000005	0.000002	0.000182	0.000122	-	-	-	-	-
YR3-3	0.000208	0.000002	0.000001	0.000003	0.000004	0.000003	-	-	-	-
YR4-4	0.000208	0.000001	0.000004	0.000002	0.000002	0.000002	0.000255	-	-	-
YR4-9	0.000208	0.000004	0.000042	0.000004	0.000005	0.000004	0.000042	0.000023	-	-
YR5-2	0.000208	0.000042	0.000002	0.000042	0.000042	0.000042	0.000208	0.000194	>0.999999	-

Table S3 Multiple t-test analysis on the k_{cat}/K_M values of TEM variants. The adjusted p-values are shown. The values shown in red indicates the p-values larger than 0.05.

	TEM	E166Y	YR1-3	YR1-5	YR2-4	YR2-5	YR3-3	YR4-4	YR4-9	YR5-2
TEM	-	-	-	-	-	-	-	-	-	-
E166Y	0.019190	-	-	-	-	-	-	-	-	-
YR1-3	0.019190	0.001737	-	-	-	-	-	-	-	-
YR1-5	0.019190	0.002566	0.022408	-	-	-	-	-	-	-
YR2-4	0.019190	0.002566	0.004168	0.008112	-	-	-	-	-	-
YR2-5	0.019190	0.001253	0.004705	0.039819	0.013995	-	-	-	-	-
YR3-3	0.020054	0.000538	0.000749	0.001114	0.022408	0.001362	-	-	-	-
YR4-4	0.023664	0.001270	0.001403	0.001591	0.003794	0.001737	0.008752	-	-	-
YR4-9	0.023529	0.000904	0.001045	0.001170	0.002940	0.001260	0.008490	0.191643	-	-
YR5-2	0.097342	0.039819	0.039819	0.039819	0.039819	0.039819	0.039819	0.048909	0.048909	-

Table S4 Selection condition and library size used in each round of directed evolution

Evolutionary Round	Selection vector	aTc concentration (nM)	Library size	% survival
1	pAT	25	6.0×10^5	0.09%
2	pAT	10	1.3×10^5	0.61%
3	pAT	2.5	1.1×10^7	0.06%
4	pAT	0	6.3×10^6	0.16%
	pAT-*cr3*	100	5.6×10^6	0.07%
5	pAT	0	1.1×10^7	0.26%
	pAT-*cr3*	50	1.4×10^7	0.09%

Table S5 Average colony diameter of *E. coli* XL1-Blue expressing evolved TEM variants under the control of the pAT vector. All selected gene variants were subcloned into pAT. Each TEM variant-expressing *E. coli* strain was grown on LB Amp50 containing varying concentrations of aTc and on LB Cm30 at 37°C for 24 h. *E. coli* XL1-Blue containing the pAT plasmid was used as the negative control. Colony diameters were determined by ImageJ[25].

TEM variant	Average colony diameter (mm)					
	aTc concentration (nM)					
	0	2.5	5	10	25	Cm
**Constitutive TEM**	1.4 ± 0.2	1.4 ± 0.2	1.5 ± 0.2	1.3 ± 0.1	1.2 ± 0.2	1.3 ± 0.1
**No TEM**	0.0 ± 0.0	0.0 ± 0.0	0.0 ± 0.0	0.0 ± 0.0	0.0 ± 0.0	1.4 ± 0.1
**pAT-TEM**	1.4 ± 0.2	1.5 ± 0.06	1.5 ± 0.2	1.5 ± 0.1	1.4 ± 0.1	1.4 ± 0.2
**pAT-E166Y**	0.0 ± 0.0	0.0 ± 0.0	0.0 ± 0.0	0.0 ± 0.0	0.0 ± 0.0	1.4 ± 0.2
**pAT-YR1-3**	0.0 ± 0.0	0.0 ± 0.0	0.5 ± 0.0	0.7 ± 0.0	0.7 ± 0.0	1.3 ± 0.06
**pAT-YR1-5**	0.0 ± 0.0	0.0 ± 0.0	0.5 ± 0.05	0.9 ± 0.05	0.9 ± 0.08	1.3 ± 0.05
**pAT-YR2-4**	0.0 ± 0.0	0.4 ± 0.2	0.9 ± 0.06	1.2 ± 0.3	1.3 ± 0.1	1.4 ± 0.1
**pAT-YR2-5**	0.0 ± 0.0	0.5 ± 0.0	1.0 ± 0.0	1.3 ± 0.2	1.2 ± 0.2	1.4 ± 0.3
**pAT-YR3-3**	1.1 ± 0.0	1.5 ± 0.06	1.9 ± 0.2	1.7 ± 0.1	1.4 ± 0.06	1.2 ± 0.1
**pAT-YR4-4**	1.1 ± 0.06	1.6 ± 0.2	1.8 ± 0.3	1.7 ± 0.1	1.8 ± 0.06	1.4 ± 0.1
**pAT-YR4-9**	1.2 ± 0.06	1.5 ± 0.0	1.7 ± 0.2	1.7 ± 0.2	1.4 ± 0.2	1.5 ± 0.3
**pAT-YR5-2**	1.3 ± 0.1	1.5 ± 0.1	1.4 ± 0.0	1.5 ± 0.06	1.4 ± 0.06	1.4 ± 0.06

Table S6 Average colony diameter of *E. coli* XL1-Blue expressing evolved TEM variants under the control of the pAT-*cr3* vector. All selected gene variants were subcloned into pAT-*cr3*. Each TEM variant-expressing *E. coli* strain was grown on LB Amp50 containing varying concentrations of aTc and on LB Cm30 at 37°C for 24 h. *E. coli* XL1-Blue containing the pAT-*cr3* plasmid was used as the negative control. Colony diameters were determined by ImageJ[25].

TEM variant	Average colony diameter (mm)					
	aTc concentration (nM)					
	0	50	100	250	500	Cm
**Constitutive TEM**	1.2 ± 0.1	1.2 ± 0.06	1.2 ± 0.1	1.2 ± 0.2	1.3 ± 0.06	1.3 ± 0.2
**No TEM**	0.0 ± 0.0	0.0 ± 0.0	0.0 ± 0.0	0.0 ± 0.0	0.0 ± 0.0	1.3 ± 0.06
**pAT-*cr3*-TEM**	0.0 ± 0.0	0.9 ± 0.1	1.5 ± 0.06	1.4 ± 0.2	1.5 ± 0.2	1.4 ± 0.2
**pAT-*cr3*-E166Y**	0.0 ± 0.0	0.0 ± 0.0	0.0 ± 0.0	0.0 ± 0.0	0.0 ± 0.0	1.4 ± 0.0
**pAT-*cr3*-YR1-3**	0.0 ± 0.0	0.0 ± 0.0	0.0 ± 0.0	0.5 ± 0.06	1.1 ± 0.1	1.4 ± 0.06
**pAT-*cr3*-YR1-5**	0.0 ± 0.0	0.0 ± 0.0	0.0 ± 0.0	0.5 ± 0.0	1.0 ± 0.0	1.5 ± 0.1
**pAT-*cr3*-YR2-4**	0.0 ± 0.0	0.0 ± 0.0	0.0 ± 0.0	0.9 ± 0.1	1.1 ± 0.06	1.5 ± 0.06
**pAT-*cr3*-YR2-5**	0.0 ± 0.0	0.0 ± 0.0	0.5 ± 0.06	0.9 ± 0.0	1.1 ± 0.06	1.4 ± 0.1
**pAT-*cr3*-YR3-3**	0.0 ± 0.0	0.0 ± 0.0	0.8 ± 0.06	1.1 ± 0.1	1.3 ± 0.0	1.4 ± 0.0
**pAT-*cr3*-YR4-4**	0.0 ± 0.0	0.5 ± 0.0	0.9 ± 0.0	1.2 ± 0.06	1.3 ± 0.0	1.5 ± 0.06
**pAT-*cr3*-YR4-9**	0.0 ± 0.0	0.5 ± 0.0	0.9 ± 0.0	1.5 ± 0.1	1.4 ± 0.0	1.5 ± 0.06
**pAT-*cr3*-YR5-2**	0.0 ± 0.0	0.8 ± 0.0	1.3 ± 0.1	1.4 ± 0.06	1.5 ± 0.2	1.3 ± 0.06

Table S7 Average colony diameter of *E. coli* XL1-Blue expressing E166Y TEM variants selected from the fourth round of directed evolution. The YR4-1 to YR4-12 variants were selected with pAT-*cr3*, while YR4-13 to YR4-24 variants were originally selected with pAT. All selected gene variants were subcloned into pAT-*cr3.* Each TEM variant-expressing *E. coli* strain was grown on LB Amp50 containing varying concentrations of aTc and on LB Cm30 at 37ºC for 24 h. *E. coli* XL1-Blue containing the pAT-*cr3* plasmid was used as the negative control. Colony diameters were determined by ImageJ[25].

TEM variant	Average colony diameter (mm)			
	aTc concentration (nM)			
	50	100	250	Cm
**Constitutive TEM**	1.4 ± 0.1	1.4 ± 0.2	1.5 ± 0.1	1.3 ± 0.2
**No TEM**	0.0 ± 0.0	0.0 ± 0.0	0.0 ± 0.0	1.3 ± 0.3
**pAT-*cr3*-YR3-3**	0.0 ± 0.0	0.7 ± 0.1	1.2 ± 0.2	1.4 ± 0.3
**pAT-*cr3*-YR4-1**	0.0 ± 0.0	0.7 ± 0.1	0.8 ± 0.3	1.4 ± 0.2
**pAT-*cr3*-YR4-2**	0.4 ± 0.0	0.9 ± 0.2	1.1 ± 0.2	1.4 ± 0.06
**pAT-*cr3*-YR4-3**	0.4 ± 0.0	0.9 ± 0.2	1.1 ± 0.3	1.4 ± 0.2
**pAT-*cr3*-YR4-4**	0.4 ± 0.06	1.0 ± 0.2	1.2 ± 0.06	1.4 ± 0.06
**pAT-*cr3*-YR4-5**	0.0 ± 0.0	0.0 ± 0.0	0.0 ± 0.0	1.3 ± 0.06
**pAT-*cr3*-YR4-6**	0.0 ± 0.0	0.7 ± 0.06	0.7 ± 0.06	1.4 ± 0.06
**pAT-*cr3*-YR4-7**	0.4 ± 0.0	1.0 ± 0.2	1.2 ± 0.2	1.5 ± 0.06
**pAT-*cr3*-YR4-8**	0.3 ± 0.06	0.8 ± 0.1	1.0 ± 0.2	1.4 ± 0.2
**pAT-*cr3*-YR4-9**	0.5 ± 0.06	1.0 ± 0.1	1.5 ± 0.1	1.5 ± 0.06
**pAT-*cr3*-YR4-10**	0.4 ± 0.0	0.8 ± 0.0	1.3 ± 0.1	1.3 ± 0.2
**pAT-*cr3*-YR4-11**	0.0 ± 0.0	0.9 ± 0.06	1.1 ± 0.06	1.4 ± 0.1
**pAT-*cr3*-YR4-12**	0.4 ± 0.06	0.9 ± 0.06	1.3 ± 0.1	1.3 ± 0.06
**pAT-*cr3*-YR4-13**	0.0 ± 0.0	0.0 ± 0.0	0.9 ± 0.3	1.3 ± 0.2
**pAT-*cr3*-YR4-14**	0.0 ± 0.0	0.0 ± 0.0	0.6 ± 0.0	1.3 ± 0.3
**pAT-*cr3*-YR4-15**	0.0 ± 0.0	0.0 ± 0.0	0.0 ± 0.0	1.4 ± 0.3
**pAT-*cr3*-YR4-16**	0.0 ± 0.0	0.0 ± 0.0	0.0 ± 0.0	1.3 ± 0.06
**pAT-*cr3*-YR4-17**	0.3 ± 0.06	1.0 ± 0.06	1.3 ± 0.1	1.3 ± 0.1
**pAT-*cr3*-YR4-18**	0.5 ± 0.0	0.7 ± 0.06	1.2 ± 0.1	1.3 ± 0.1
**pAT-*cr3*-YR4-19**	0.4 ± 0.0	0.9 ± 0.06	1.4 ± 0.2	1.3 ± 0.06
**pAT-*cr3*-YR4-20**	0.0 ± 0.0	0.0 ± 0.0	0.0 ± 0.0	1.2 ± 0.2
**pAT-*cr3*-YR4-21**	0.4 ± 0.06	0.8 ± 0.0	1.3 ± 0.2	1.4 ± 0.2
**pAT-*cr3*-YR4-22**	0.4 ± 0.06	0.9 ± 0.1	1.3 ± 0.06	1.4 ± 0.2
**pAT-*cr3*-YR4-23**	0.0 ± 0.0	0.0 ± 0.0	0.0 ± 0.0	1.4 ± 0.0
**pAT-*cr3*-YR4-24**	0.0 ± 0.0	0.0 ± 0.0	0.0 ± 0.0	1.3 ± 0.1

Table S8 Average colony diameter of *E. coli* XL1-Blue expressing E166Y TEM variants selected from the fifth round of directed evolution. The YR5-1 to YR5-12 variants were selected with pAT-*cr3*, while YR5-13 to YR5-24 variants were originally selected with pAT. All selected gene variants were subcloned into pAT-*cr3.* Each TEM variant-expressing *E. coli* strain was grown on LB Amp50 containing varying concentrations of aTc and on LB Cm30 at 37°C for 24 h. *E. coli* XL1-Blue containing the pAT-*cr3* plasmid was used as the negative control. Colony diameters were determined by ImageJ[25].

TEM variant	Average colony diameter (mm)			
	aTc concentration (nM)			
	50	100	250	Cm
**Constitutive TEM**	1.4 ± 0.1	1.4 ± 0.2	1.5 ± 0.1	1.3 ± 0.2
**No TEM**	0.0 ± 0.0	0.0 ± 0.0	0.0 ± 0.0	1.3 ± 0.3
**pAT-*cr3*-YR4-4**	0.4 ± 0.06	1.0 ± 0.2	1.2 ± 0.06	1.4 ± 0.06
**pAT-*cr3*-YR4-9**	0.5 ± 0.06	1.0 ± 0.1	1.5 ± 0.1	1.5 ± 0.06
**pAT-*cr3*-YR5-1**	0.0 ± 0.0	0.0 ± 0.0	0.0 ± 0.0	1.3 ± 0.2
**pAT-*cr3*-YR5-2**	0.8 ± 0.0	1.0 ± 0.06	1.2 ± 0.06	1.5 ± 0.2
**pAT-*cr3*-YR5-3**	0.5 ± 0.0	1.2 ± 0.06	1.2 ± 0.0	1.5 ± 0.2
**pAT-*cr3*-YR5-4**	0.5 ± 0.0	0.9 ± 0.06	1.2 ± 0.1	1.4 ± 0.1
**pAT-*cr3*-YR5-5**	0.5 ± 0.1	1.0 ± 0.2	1.1 ± 0.2	1.5 ± 0.2
**pAT-*cr3*-YR5-6**	0.5 ± 0.0	1.1 ± 0.06	1.2 ± 0.0	1.3 ± 0.1
**pAT-*cr3*-YR5-7**	0.5 ± 0.0	1.1 ± 0.06	1.3 ± 0.0	1.5 ± 0.2
**pAT-*cr3*-YR5-8**	0.7 ± 0.0	1.3 ± 0.0	1.4 ± 0.2	1.4 ± 0.2
**pAT-*cr3*-YR5-9**	0.7 ± 0.0	1.2 ± 0.0	1.3 ± 0.2	1.6 ± 0.1
**pAT-*cr3*-YR5-10**	0.5 ± 0.0	1.2 ± 0.06	1.3 ± 0.06	1.5 ± 0.3
**pAT-*cr3*-YR5-11**	0.5 ± 0.0	1.1 ± 0.2	1.2 ± 0.1	1.4 ± 0.2
**pAT-*cr3*-YR5-12**	0.5 ± 0.0	1.0 ± 0.2	1.1 ± 0.06	1.4 ± 0.2
**pAT-*cr3*-YR5-13**	0.0 ± 0.0	0.0 ± 0.0	0.5 ± 0.06	1.4 ± 0.1
**pAT-*cr3*-YR5-14**	0.0 ± 0.0	0.0 ± 0.0	0.7 ± 0.06	1.4 ± 0.06
**pAT-*cr3*-YR5-15**	0.0 ± 0.0	0.0 ± 0.0	0.0 ± 0.0	1.6 ± 0.2
**pAT-*cr3*-YR5-16**	0.0 ± 0.0	0.0 ± 0.0	0.5 ± 0.0	1.3 ± 0.06
**pAT-*cr3*-YR5-17**	0.0 ± 0.0	0.6 ± 0.0	0.9 ± 0.06	1.4 ± 0.1
**pAT-*cr3*-YR5-18**	0.0 ± 0.0	0.0 ± 0.0	0.0 ± 0.0	1.3 ± 0.2
**pAT-*cr3*-YR5-19**	0.0 ± 0.0	0.5 ± 0.0	0.9 ± 0.0	1.5 ± 0.2
**pAT-*cr3*-YR5-20**	0.0 ± 0.0	0.6 ± 0.0	0.9 ± 0.0	1.3 ± 0.2
**pAT-*cr3*-YR5-21**	0.0 ± 0.0	0.5 ± 0.0	0.9 ± 0.0	1.4 ± 0.1
**pAT-*cr3*-YR5-22**	0.0 ± 0.0	0.0 ± 0.0	0.0 ± 0.0	1.6 ± 0.2
**pAT-*cr3*-YR5-23**	0.0 ± 0.0	0.6 ± 0.06	1.0 ± 0.0	1.5 ± 0.1
**pAT-*cr3*-YR5-24**	0.0 ± 0.0	0.0 ± 0.0	0.6 ± 0.1	1.5 ± 0.2

Table S9 Mutations in the evolved mutants selected from the fifth round of directed evolution. The E166Y mutation is highlighted in purple. Other mutations are highlighted in pink. Residue numbering is based on the Ambler numbering scheme[35].

TEM variant	Amino acid residue																									Number of mutations
	4	20	31	60	68	103	133	140	160	166	170	172	182	184	188	191	192	198	209	214	217	240	241	273	285	
WT	S	C	V	F	M	V	T	T	T	E	N	I	M	V	T	R	K	L	D	D	A	E	R	R	L	0
E166Y	S	C	V	F	M	V	T	T	T	Y	N	I	M	V	T	R	K	L	D	D	A	E	R	R	L	1
YR5-2	T	C	M	F	I	V	S	T	T	Y	S	I	T	V	T	R	K	V	D	N	T	G	H	R	L	12
YR5-8	R	C	V	F	M	V	S	R	T	Y	S	K	T	V	I	R	K	V	D	N	T	G	H	R	L	13
YR5-9	S	C	V	Y	M	L	T	T	S	Y	S	I	T	V	I	R	K	V	D	N	T	G	H	R	M	13

Table S10 Primers used in this study

Primer	Sequence	Annealing temperature (°C)
AvrII-Cm-F	5’-ATACC TAGGT GATCG GCACG TAAGA GG-3’	66
AvrII-KTR-R	5’-TTACC TAGGG TTTTC CCAGT C-3’	61
Cis-F	5’-AATCG TAATG CGTCG ATAGA AGGAG AGGTT C-3’	61
E166Y-F	5’-CTTGA TCGTT GGTAC CCGGA GCTGA ATGAA GC-3’	68
E166Y-R	5’-CAGCT CCGGG TACCA ACGAT CAAGG CGAG-3’	65
EcoRI-pAmp-F	5’-ACTGA ATTCG CGGAA CCCCT ATTTG-3’	65
HindIII-pAT-R	5’-TATAA GCTTG CAAAA GGCCA GCAAA AG-3’	65
HindIII-tetR-F	5’-CCTTT TGCTC AAGCT TTTAA GACCC ACTTT CACAT TTAAG TTG-3’	72
NdeI-Amp-F	5’-CCGCA TATGA GTATT CAACA TTTCC G-3’	65
NdeI-cis-R	5’-ACTCA TATGT CGAAC CTCTC CTTCT ATCGA CGCAT TACGA TT-3’	72
PacI-Cm-R	5’-TTAAT TAATT ACGCC CCGCC CTG-3’	66
PacI-KTR-F	5’-TTAAT TAACT GTCAG ACCAA GTTTA C-3’	61
pET-F	5’-TACCA TATGC ACCCA GAAAC GCTGG TG-3’	68
pET-V31M-F	5’-TACCA TATGC ACCCA GAAAC GCTGA TG-3’	68
T7 promoter	5’-TAATA CGACT CACTA TAGGG-3’	56
XhoI-Amp-R	5’-ATTAC TCGAG CCAAT GCTTA ATCAG TGAG-3’	68
